# The continuum of *Drosophila* embryonic development at single-cell resolution

**DOI:** 10.1126/science.abn5800

**Published:** 2022-08-05

**Authors:** Diego Calderon, Ronnie Blecher-Gonen, Xingfan Huang, Stefano Secchia, James Kentro, Riza M. Daza, Beth Martin, Alessandro Dulja, Christoph Schaub, Cole Trapnell, Erica Larschan, Kate M. O’Connor-Giles, Eileen E. M. Furlong, Jay Shendure

**Affiliations:** 1 Department of Genome Sciences, University of Washington, Seattle, WA 98195, USA; 2 The Crown Genomics Institute of the Nancy and Stephen Grand Israel National Center for Personalized Medicine, Weizmann Institute of Science, Rehovot, Israel; 3 Paul G. Allen School of Computer Science & Engineering, University of Washington, Seattle, WA 98195, USA; 4 European Molecular Biology Laboratory (EMBL), Genome Biology Unit, Heidelberg, Germany; 5 Molecular Biology, Cell Biology, and Biochemistry Graduate Program, Brown University, Providence, RI 02912, USA; 6 Brotman Baty Institute for Precision Medicine, University of Washington, Seattle, WA 98195, USA; 7 Allen Discovery Center for Cell Lineage Tracing, Seattle, WA 98195, USA; 8 Department of Neuroscience and Carney Institute for Brain Science, Brown University, Providence, RI 02912, USA; 9 Howard Hughes Medical Institute, University of Washington, Seattle, WA 98195, USA

## Abstract

**INTRODUCTION:**

Single-cell technologies are a powerful means of studying metazoan development, enabling comprehensive surveys of cellular diversity at profiled time points and shedding light on the dynamics of regulatory element activity and gene expression changes during the in vivo emergence of each cell type. However, nearly all such whole-embryo atlases of embryogenesis remain limited by sampling density—i.e., the number of discrete time points at which individual embryos are harvested and cells or nuclei are collected. Given the rapidity with which molecular and cellular programs unfold, this limits the resolution at which regulatory transitions can be characterized. For example, in the mouse, there are typically *6* to 24 hours between sampled embryonic time points—gaps within which massive molecular and morphological changes take place.

**RATIONALE:**

To construct an ungapped representation of embryogenesis in vivo, we would ideally sample embryos continuously. Although this is not practical for most model organisms, it is potentially possible in *Drosophila melanogaster,* where collections of timed and yet somewhat asynchronous embryos are easy to obtain, such that, at least in principle, one can achieve arbitrarily high temporal resolution. *Drosophila* could therefore serve as a test case to develop a framework for the inference of continuous regulatory and cellular trajectories of in vivo embryogenesis. Because *Drosophila* is a preeminent model organism that has yielded many advances in the biological and biomedical sciences, obtaining a single-cell atlas of *Drosophila* embryogenesis is also an important goal in itself. This includes its embryonic development, where the use of this model in conjunction with powerful genetic tools has transformed our understanding of the mechanisms by which developmental complexity is achieved, in addition to uncovering many general principles of both genetic and epigenetic gene regulation.

**RESULTS:**

We profiled chromatin accessibility in almost 1 million nuclei and gene expression in half a million nuclei from eleven overlapping windows spanning the entirety of embryogenesis (0 to 20 hours). To exploit the developmental asynchronicity of embryos from each collection window, we applied deep neural network-based predictive modeling to more-precisely predict the developmental age of each nucleus within the dataset, resulting in continuous, multimodal views of molecular and cellular transitions in absolute time. With these data, the dynamics of enhancer usage and gene expression can be explored within and across lineages at the scale of minutes, including for precise transitions like zygotic genome activation.

**CONCLUSION:**

This *Drosophila* embryonic atlas broadly informs the orchestration of cellular states during the most dynamic stages in the life cycle of metazoan organisms. The inclusion of predicted nuclear ages will facilitate the exploration of the precise time points at which genes become active in distinct tissues as well as how chromatin is remodeled across time.

## Abstract (summary)

*Drosophila melanogaster* is a powerful, long-standing model for metazoan development and gene regulation. We profiled chromatin accessibility in almost 1 million and gene expression in half a million nuclei from overlapping windows spanning the entirety of embryogenesis. Leveraging developmental asynchronicity within embryo collections, we applied deep neural networks to infer the age of each nucleus, resulting in continuous, multimodal views of molecular and cellular transitions in absolute time. We identify cell lineages; infer their developmental relationships; and link dynamic changes in enhancer usage, transcription factor (TF) expression, and the accessibility of TFs’ cognate motifs. With these data, the dynamics of enhancer usage and gene expression can be explored within and across lineages at the scale of minutes, including for precise transitions like zygotic genome activation.

## Graphical Abstract

**Characterizing the continuum of *Drosophila* embryogenesis.** We collected staged *Drosophila* embryos from overlapping time windows across the first 20 hours of embryogenesis. Then we extracted nuclei and performed single-cell RNA sequencing (RNA-seq) and assay for transposase-accessible chromatin using sequencing (ATAC-seq) profiling using combinatorial indexing (sci-RNA-seq and sci-ATAC-seq) to comprehensively map expressed genes and putatively active regulatory elements. We applied machine learning to infer a continuum of nuclear ages that is synchronized across unfolding lineages in absolute time. The continuous nuclear age predictions were used to annotate and then link cellular states at nonoverlapping 2-hour intervals, as well as to explore transcriptional regulatory dynamics across major cell lineages of embryonic development at fine-scale temporal resolution.

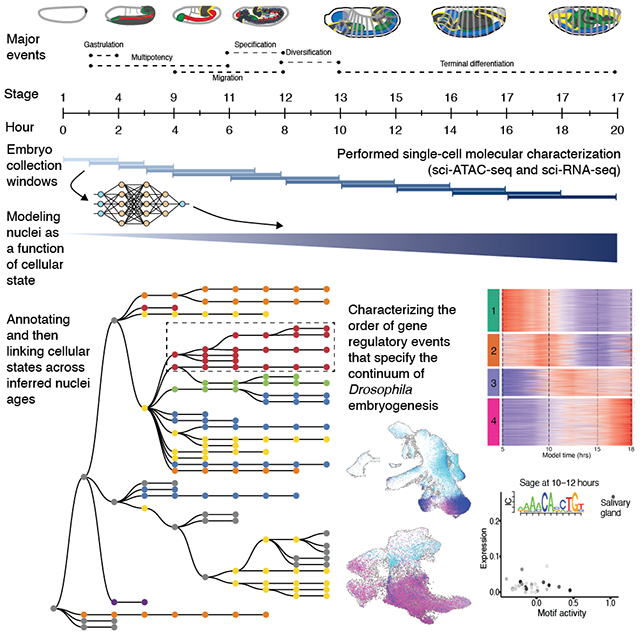

Single-cell technologies are a powerful means of studying metazoan development, shedding light on the emergence of cellular diversity and the dynamics of gene regulation. However, nearly all such atlases of embryogenesis are limited in terms of the number of discrete time points and cells sampled per time point. Given the rapidity with which molecular and cellular programs unfold, this limits the resolution at which regulatory transitions can be characterized.

To more completely represent development, embryos would ideally be sampled continuously. Although impractical for most model organisms, it is feasible in *Drosophila,* where collections of timed and yet somewhat asynchronous embryos are easy to obtain, such that, in principle, one can achieve arbitrarily high temporal resolution. This sharply contrasts with mice, for which there are typically 6 to 24 hours between sampled time points, gaps within which massive molecular and morphological changes take place (1–4). Although sampling gaps can be computationally filled through the continuum of cell states represented in single embryos (4, 5), the asynchronous ages of *Drosophila* embryos within staged collections present an opportunity for more bona fide continuity—e.g., with seconds or minutes separating the developmental ages of consecutive embryos rather than hours or days. Moreover, because *Drosophila melanogaster* is a preeminent model organism that has yielded many discoveries and general principles of metazoan development and gene regulation, obtaining a single-cell atlas of *Drosophila* embryogenesis is an important goal in itself.

## Results

**Fig. 1.**
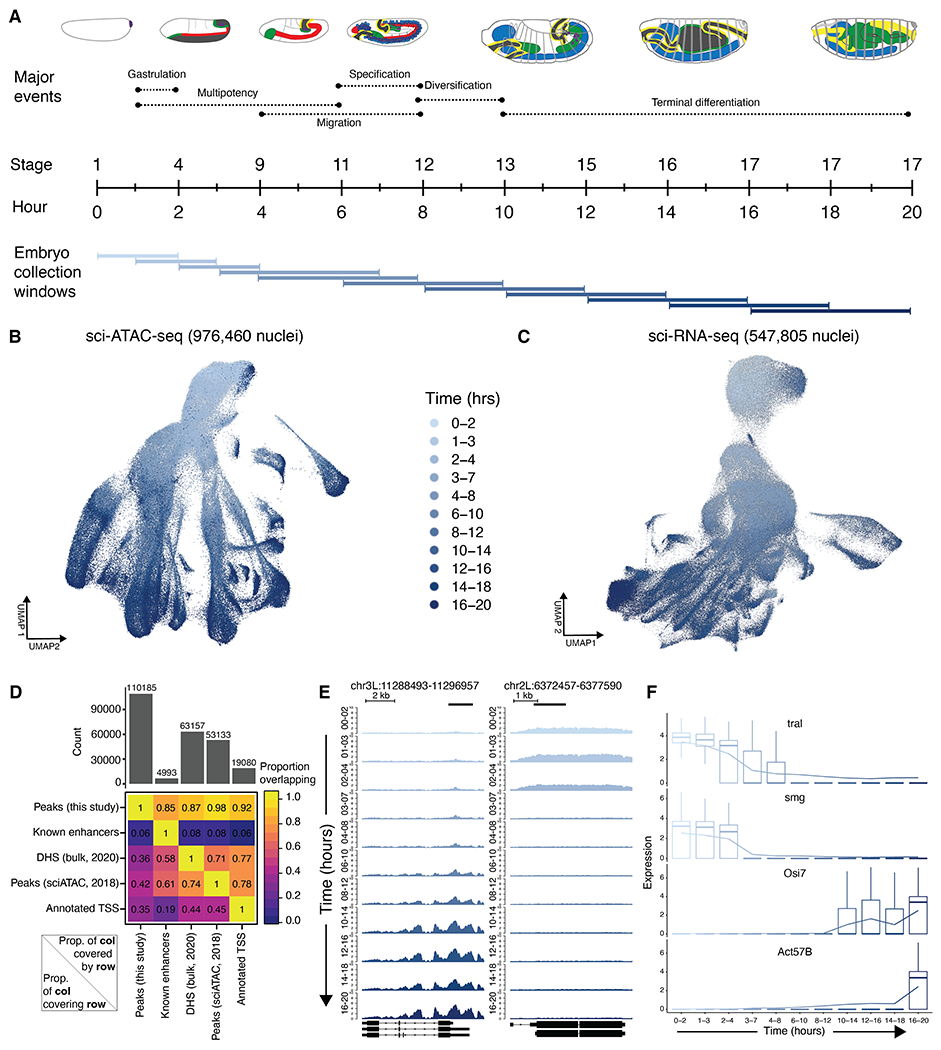
Single-cell profiling of chromatin accessibility and gene expression throughout *Drosophila* embryogenesis. (**A**) Eleven overlapping collection windows that collectively cover embryogenesis. (**B**) UMAP visualization of cell-x-peak matrix of evenly time-subsampled sci-ATAC-seq nuclei that passed QC. (**C**) Same as (B), but for sci-RNA-seq. (**D**) Heatmap showing proportion of our scATAC peaks overlapping ~5000 curated enhancers (8–10), bulk DHS peaks from 2 to 12 hours (11), scATAC peaks from 2 to 12 hours (12), or annotated TSSs (49). (**E**) Chromatin accessibility, normalized by counts per million reads, across representative regions exhibiting time dependence across 11 collection windows. (**F**) Gene expression of representative genes exhibiting time dependence across 11 collection windows. Read counts were normalized, multiplied by a scale-factor, log-transformed after the addition of a pseudocount, and averaged across all cells within each window.

We set out to measure chromatin accessibility and gene expression from individual nuclei spanning a continuum of *D. melanogaster* embryogenesis. Staged embryos were collected in 11 overlapping time windows, collectively 0 to 20 hours, covering the entirety of embryogenesis at 25°C. Overlapping 2-hour collections were used to capture the rapid transitions during early stages, followed by overlapping 4-hour collections from 3 hours onward (Fig. 1A). From each collection, samples were split and separately processed for assay for transposase-accessible chromatin using sequencing (ATAC-seq) or RNA sequencing (RNA-seq). Although we hereafter refer to cells, all data were generated from nuclei. Single-cell profiling was conducted using three-level combinatorial indexing (sci-ATAC-seq3 and sci-RNA-seq3) with minor modifications (1, 6).

Sci-ATAC-seq3 and sci-RNA-seq3 libraries were sequenced to generate 30 billion and 6.8 billion raw reads, respectively (fig. S1). After deduplication and application of quality filters, we obtained chromatin accessibility profiles for 976,460 cells [single-cell ATAC (scATAC): median 5206 nonduplicate reads per cell] and gene expression profiles for 547,805 cells [single-cell RNA (scRNA): median 399 unique molecular identifiers (UMIs) and 274 genes detected per cell]. Although our scRNA data have fewer UMIs per nucleus than previously obtained from *Drosophila* embryos (7), we profiled many more nuclei spanning many more stages of embryogenesis and complemented this with scATAC with a high number of unique reads per nucleus. Given the small size of the *Drosophila* embryo, such deep “shotgun cellular coverage” should effectively sample all tissue types during embryogenesis. The data did not appear to be confounded by batch effects (fig. S2, A to G).

For both data modalities, integrating and visualizing single-cell profiles across all time points resulted in branching structures going from early to late stages, consistent with increasing complexity (Fig. 1, B and C). From the scATAC data, we identified 110,185 regions exhibiting accessibility at some point during embryogenesis. Collectively, these candidate regulatory elements cover 30.4 Mb (22%) of *Drosophila* euchromatin (dm6) and include 85% of known embryonic enhancers, based on overlap with nearly 5000 curated enhancers confirmed in transgenic embryos (Fig. 1D) (8–10). This, together with the high coverage of both bulk deoxyribonuclease (DNase) I hypersensitive site (DHS) peaks (87%) and scATAC-derived peaks (98%) from 2 to 12 hours (11, 12), supports the comprehensiveness of this compendium. Similar results were obtained computing overlaps on a per-base rather than per-element basis (fig. S2H). We additionally uncovered more than 40,000 distal accessible regions not identified in these previous studies (Fig. 1D) that are enriched for enhancer-associated histone marks, suggesting that they are previously uncharacterized developmental enhancers (fig. S2I). The compendium also recovered 94% of 8008 extensively validated mesodermal cis-regulatory modules (13) and 96% of nearly 1 million chromatin immunoprecipitation (ChIP)–defined binding sites across 233 transcription factors (TFs) (14) (fig. S2J).

In exploring these data, we identified thousands of genomic regions and transcripts whose accessibility and expression levels, respectively, were strongly correlated with the progression of developmental time (Fig. 1, E and F). Notably, not all of these correlations were cell type specific (fig. S3). The presence of such time-dependent elements and transcripts suggests that a dynamic process is unfolding across development, at least some aspects of which are cell type specific, whereas other aspects appear general to germ layers or the entire organism. We reasoned that we could leverage these correlations to build a model to predict absolute developmental age of any given nucleus with greater temporal resolution than our 2- to 4-hour collection windows.

### Predicting the absolute age of individual nuclei

**Fig. 2.**
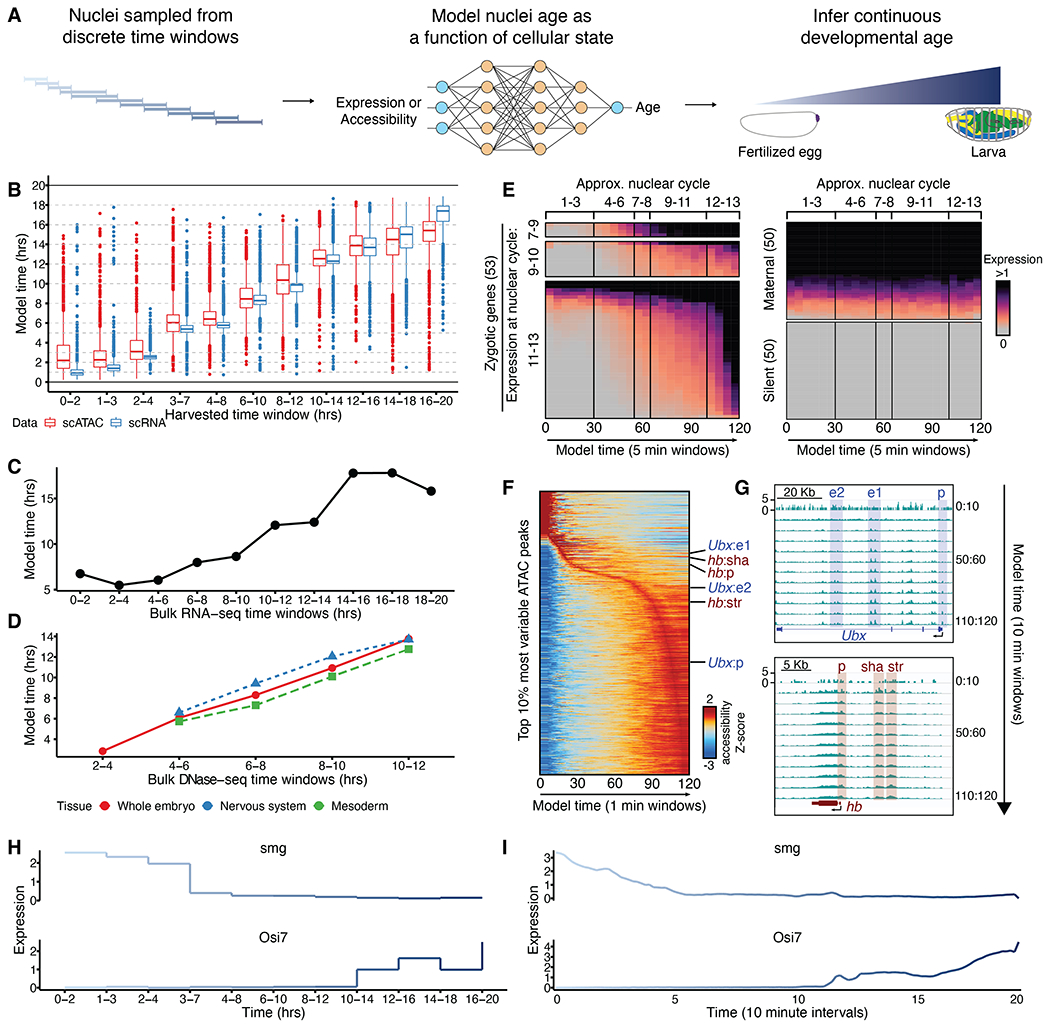
Inferring developmental age from cellular state. (**A**) We fit a NN-based model that uses either gene expression or chromatin accessibility to predict the center hour of the time window from which each nucleus was sampled. The inferred nuclear ages make up a continuum. (**B**) NN model–predicted developmental ages (*y* axis) of test set nuclei, equally sampled from discrete time windows (*x* axis) and not included in model training. (**C**) NN model–predicted developmental ages (*y* axis) of bulk RNA-seq samples (15) collected from 2-hour windows (*x* axis). (**D**) NN model–predicted developmental ages (*y* axis) of bulk DNase-seq samples from either whole-embryo or purified tissues collected from 2-hour windows (*x* axis). (**E**) Expression of zygotic (left), maternal (top right), or silent (bottom right) genes in nuclei from predicted age windows in 5-min increments across 0 to 2 hours of development. (**F**) Accessibility of most variable scATAC peaks from predicted age windows in 1-min increments across 0 to 2 hours of development. Labels indicate regions illustrated in (G). (**G**) Examples of cis-regulatory regions known to exhibit dynamic accessibility in early embryos (17). (**H** and **I**) Examples of time-associated genes, with expression values averaged across all nuclei from indicated collection windows (H) or from predicted age windows in 10-min increments (I).

In these data, the precise developmental age of each sampled nucleus is unknown—only the 2- to 4-hour collection window from which it derived. To estimate the age of each nucleus with greater precision, we fit a series of models using either the scATAC or scRNA data as input and predicting the center hour of the collection window from which any given nucleus was obtained (Fig. 2A). Specifically, we split a subset of each dataset, evenly subsampled with respect to time, into 11 partitions, 10 of which were used as training data to fit either a lasso linear (LL) model or a neural network (NN)–based model with 10-fold cross-validation across various test parameters. After selecting the highest performing parameterization, the NN-based models markedly outperformed LL models for both data types in predicting the developmental age of nuclei within the held-out 11th partition [for NN versus LL, mean squared error (MSE): ATAC = 5.26 versus 8.8, RNA = 2.54 versus 4.72; proportion correct: ATAC = 0.67 versus 0.53, RNA = 0.87 versus 0.65]. We therefore moved forward with NN-based nuclear age predictions for the remainder of this study (Fig. 2B and fig. S4). Notably, the scRNA-based model was slightly more accurate than the scATAC-based model, likely leading to slightly older age predictions during early collection windows and slightly younger age predictions during late collection windows for scATAC ages compared with scRNA ages.

To further assess accuracy, we applied the scRNA-derived models to a bulk RNA-seq time course of staged embryos in 2-hour intervals (15) and found high concordance between predicted and actual developmental age (Fig. 2C). The scATAC-derived models were similarly able to order a time course of bulk DNase sequencing (DNase-seq) data from either whole embryos or specific fluorescence-activated cell sorting (FACS)–purified lineages (11) (Fig. 2D). To assess predicted ages at much finer time scales (minutes rather than hours), we focused on genes whose expression is activated at specific nuclear cycles during zygotic genome activation (ZGA) (16). Genes turning on during ZGA were dynamically up-regulated in association with predicted nuclear ages (scRNA-based; 5-min increments), whereas maternal and silent genes were not (Fig. 2E). Early dynamically accessible enhancers and promoters could similarly be predicted (scATAC-based; 1-min increments) (Fig. 2F), opening in the same order as previously observed by bulk ATAC-seq of hand-picked embryos at 3-min intervals (Fig. 2G) (17). To further illustrate the value of this framework, we note that pseudobulk profiles corresponding to collection windows lead to piecewise expression dynamics (Fig. 2H). By contrast, pseudobulk profiles based on model-predicted ages yield more continuous dynamics (Fig. 2I).

Although there are similarities between the goal of our approach and the concept of pseudotime (18), a key advantage of inferred age is that, both in training and prediction, cells are anchored to absolute time, which enables more interpretable ordering of cellular processes as well as their synchronization across lineages. One concern is that contamination with embryos whose developmental age falls outside the collection window will have exaggerated confounding effects on early time points because older embryos contain vastly more nuclei. Consistent with this, our model predicted that 2.8% of the ~80,000 scRNA-profiled cells from 0 to 2 hours were at least 4 hours in developmental age. These older cells represent the majority of a discrete cluster in uniform manifold approximation and projection (UMAP) space (fig. S5A). Similar contamination is also observed with scATAC profiles from this early time window (12.7% of ~20,000 cells; fig. S5, B to D). Clustering and visualizing only the cells inferred to be 0 to 2 hours in age eliminates this developmentally advanced cluster (fig. S5E).

### Annotation and inference of diversifying developmental trajectories

**Fig. 3.**
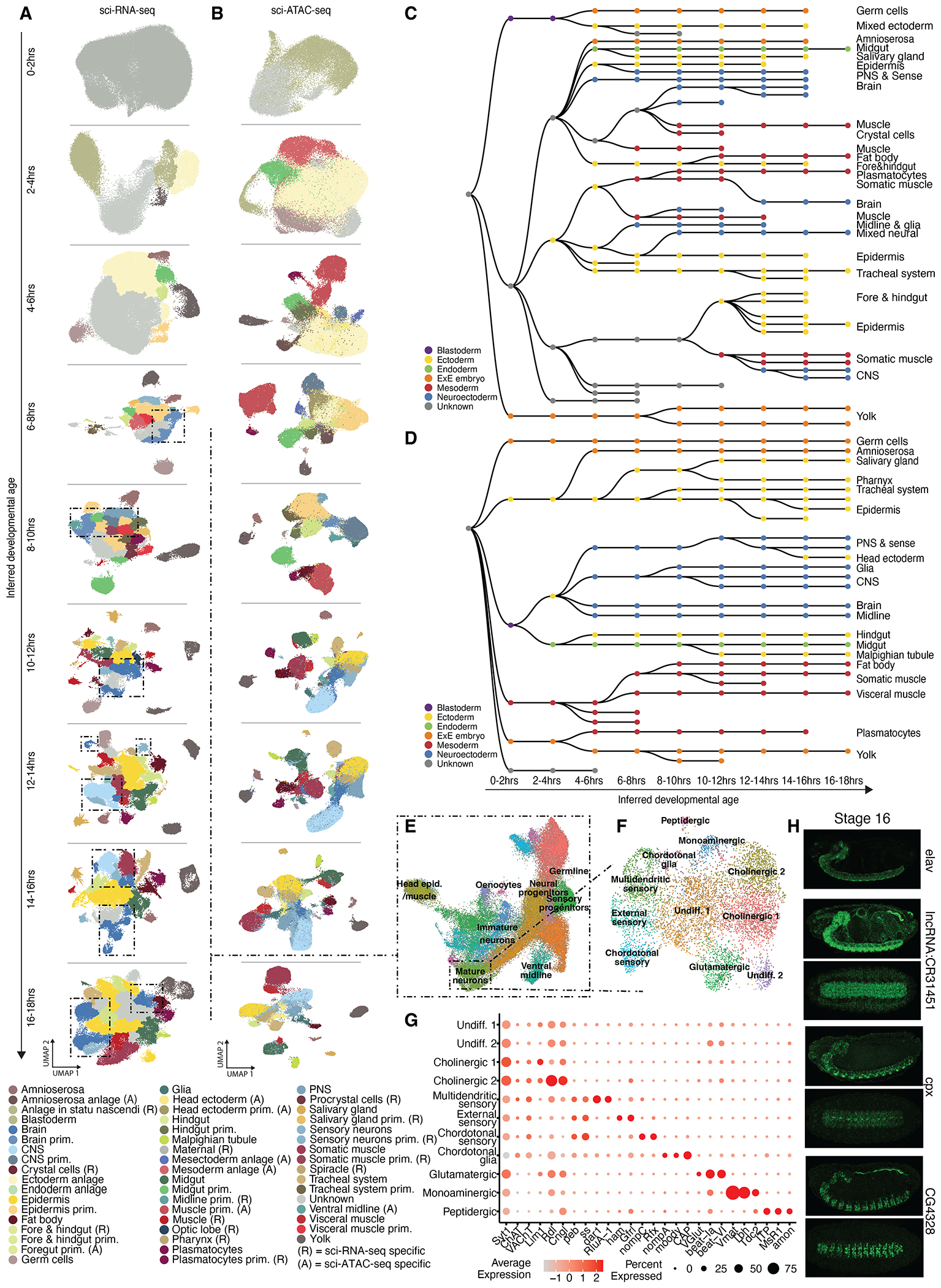
Annotation of diversifying developmental trajectories. (**A**) UMAP visualization of non-overlapping, inferred 2-hour time windows for scRNA clusters colored by cell state annotation. Dashed boxes highlight neuroectodermal clusters. (**B**) Same as (A), but for scATAC data. PNS, peripheral nervous system; CNS, central nervous system. (**C**) ScRNA-based acyclic directed graph representation of clusters linked through nonoverlapping time windows. (**D**) Same as (C), but from scATAC data. (**E**) UMAP of scRNA data for ~60,000 annotated neuroectodermal cells—i.e., cell states highlighted in (A) with dashed boxes, colored by cluster. (**F**) UMAP of ~6000 mature neurons, colored by cluster. The chordotonal glia cluster includes Ch and ES organ glial-like support cells. (**G**) Dot plot showing marker gene expression for annotated clusters in (F). (**H**) In situ hybridization of stage 16 embryos, showing the expression of lncRNA *CR31451, cpx*, and *CG4328* in the nervous system. A tissue marker (*elav*) is provided in the top panel. A lateral and ventral embryo view is shown for each gene.

To systematically track the emergence and diversification of developmental trajectories, we used inferred ages to separately process and cluster cells from a series of 2-hour nonoverlapping time windows. Clusters were then annotated by leveraging stage-matched information on gene expression from thousands of in situ hybridizations spanning embryogenesis as well as extensive enhancer activity data (12, 19, 20) (Fig. 3, A and B).

Notably, the last few hours of the time course had reduced numbers of inferred cells (e.g., after 18 hours, 61% fewer than would be expected under uniform sampling) and fewer identified clusters (fig. S6A). We suspect that this may be the result of edge effects of the model because we also observe reduced numbers of inferred cells for the first several hours, although there they have less effect because the data from early time points lack extensive structure. For this reason, we excluded cells with an inferred age of >18 hours from this set of analyses.

Here, we use cell state to mean an annotated cluster at a given time window. Altogether, we identified 171 cell states in sci-ATAC-seq data and 268 in sci-RNA-seq data across the nine time windows, each of which received one of 38 cell type annotations for ATAC or one of 54 cell type annotations for RNA (tables S1 and S2 and Fig. 3, A and B). Across time windows, we identified an average of 109 marker genes and 2469 marker accessible regions per cluster (tables S3 and S4).

The early stages of *Drosophila* embryogenesis, represented by our 0- to 2-hour time window, include 13 rapid nuclear divisions within a syncytium that generates 6000 nuclei, regulated by maternal genes. At ~2 hours and 20 min after fertilization, cellularization occurs and the zygotic genome is activated (21), followed by gastrulation to generate the three germ layers. Our single-cell data recapitulate these events, where the earliest time window (0 to 2 hours) has two large clusters annotated as maternal or unknown. At 2 to 4 hours, the maternal cluster is no longer present, and instead, pole cells and anlage clusters appear. A notable expansion in the diversity of cell types follows across 6 to 10 hours, matching expectations for when the major lineages in each germ layer are specified (Fig. 3, A and B).

To follow the emergence and diversification of cell lineages, we systematically linked cell clusters across developmental time, applying similar methods as in earlier studies (3, 22) to coembeddings of cells from adjacent nonoverlapping, inferred time windows (fig. S6, B and C). For cells of each state derived from the “child” time window, we calculated the median proportion of nearest neighbors from the “parent” window that were derived from each potential parental cell state and treated this as the weight of the corresponding edge. The maximum edge weights >0.2 were retained, resulting in acyclic, directed graphs, independently generated from scRNA and scATAC data (Fig. 3, C and D). Although these procedures were generated independently of our cell cluster annotations at each time window, they overwhelmingly yielded internally consistent results. For example, muscle clusters in one time window connect to muscle clusters in the next time window, and the same is true for other major lineages (e.g., central nervous system, peripheral nervous system, etc.) as embryogenesis proceeds. We note that some paths seem to terminate prematurely, potentially because of drastic increases in cell number in later embryogenesis, which were not matched by corresponding increases in our sampling, or because of unknown technical or biological factors. More generally, because these are inferences based on cellular state rather than lineage tracing, they may be prone to certain kinds of error (3).

To illustrate the potential of these data to facilitate exploration of specific lineages at finer resolution, we reanalyzed 59,012 cells annotated as neuroectoderm using scRNA data from 6 to 18 hours (Fig. 3E and fig. S7A). This revealed 20 subclusters, including a large group of early cells corresponding to the brain primordium and neural progenitors that express regulators of neurogenesis, such as *Notch* (*N*) and *Delta* (*Dl*), and neuroblast temporal TFs, such as *miranda* (*mira*) and *castor* (*cas*). Two additional neural progenitor clusters correspond to sensory progenitors, whereas immature neurons express low levels of both neural progenitor and pan-synaptic genes, including *cacophony* (*cac*) and *synaptotagmin 1* (*syt1*). Mature neurons are marked by higher levels of pan- and subtype-specific synaptic genes coupled with low or no expression of earlier developmental genes. Finally, midline cells, consisting of both neurons and glia cluster together, become evident at 6 to 8 hours; using the midline TF *single minded* (*sim*) and glial immunoglobulin family member *wrapper* as markers, we can follow them forward in time as they mature (fig. S7B). We can also follow the maturation of sensory neural progenitors, marked by *shaven* (*sv*), from 6 to 16 hours (fig. S7B).

To further explore neuronal diversity, we reclustered 6703 mature neurons, revealing 11 neuronal subtypes, which we manually curated (Fig. 3F). Among these, we identify four clearly separable sensory cell clusters. There are two types of *Drosophila* sensory neurons based on dendritic morphology: type I sensilla, which include both external sensory (ES) neurons and internal chordotonal (Ch) neurons, and type II multidendritic (MD) neurons. We can clearly distinguish MD neurons on the basis of expression of genes, such as dendritic arbor reduction 1 (*dar1*), which promotes their characteristic branching dendrites, and the pseudouridine synthase RluA-1, which was recently identified as a marker of MD neurons (23) (Fig. 3, F and G). Consistent with their nociceptive role, this cluster also specifically expresses the mechanical nociception degenerin/epithelial sodium channel subunits *pickpocket* (*ppk*) and *ppk26*. Mechanosensory ES neurons are specified by the TF *hamlet* (*ham*), which is specifically expressed in the middle sensory cluster (Fig. 3, F and G) (24). The adjacent cluster, likely Ch sensory neurons, is identified by expression of the mechanosensitive nonselective cation channel subunit *no mechanoreceptor potential C* (*nompC*) as well as fate-determinant *Rfx* and a number of as-yet uncharacterized genes specific to this cluster (25, 26) (Fig. 3, F and G). The final sensory cluster likely corresponds to Ch glial-like support cells based on the expression of glial markers, including *moody,* and *Cbl-associated protein* (*CAP*) and *nompA,* which promote the development and function of Ch support cells, respectively (Fig. 3, F and G). On the basis of vesicular neurotransmitter transporter expression, we also identify two clusters of central cholinergic neurons, a glutamatergic cluster that likely includes motor neurons, and monoaminergic neurons (Fig. 3, F and G). Finally, peptidergic neurons cluster separately and were identified on the basis of the expression of neuropeptides [*ion transport peptide* (*ITP*)], enzymes involved in their synthesis [*amontillado* (*amon*)], and receptors [*myosuppressin receptor 1* (*MsR1*)] (Fig. 3, F and G).

We validated the expression of uncharacterized long noncoding RNA (lncRNA) CR31451 as enriched in mature neurons as well as two genes, *complexin* (*cpx*) and *CG4328,* identified in our analysis as enriched in the monoaminergic cluster, which includes midline neurons (Fig. 3H). This neuronal subtype enrichment is unexpected for *cpx,* which encodes a presynaptic regulator of synaptic vesicle release, and may point to additional requirements for Cpx in midline monoaminergic neurons. In the course of exploring these fine neuronal subtypes, we also made an unexpected finding regarding *elav,* a classic marker gene for neurons. Specifically, we noticed lower-level expression of *elav* in clusters annotated as visceral muscle. Performing double fluorescent in situ hybridization with a visceral muscle–specific marker gene (*biniou*) confirmed this unexpected finding (fig. S7C) and raises the possibility of a potential previously unknown role of this well-studied gene.

This deeper exploration of the neuroectoderm, validating and extending years of research from many groups, illustrates the depth of information that can be obtained from these data. We additionally performed a more detailed annotation of nonmyogenic mesoderm (supplementary note 1). A full exploration of all lineages represented in these data will require a community-wide effort by tissue experts (as done in this study for neuronal diversity).

In addition to delineating developmental trajectories, these data can also capture spatial differences arising during developmental patterning. Previous bulk ATAC-seq on embryo halves has shown variability in the accessibility of enhancers along the anterior-posterior (A-P) axis of the blastoderm embryo (27). Using label transfer to map anterior or posterior identities from a previous blastoderm dataset (12) onto our 2- to 4-hour data, we computed a positional accessibility skew score for validated enhancers with strict A-P activity (27). This indicates that accessibility of most A-P enhancers is skewed in the expected anterior or posterior cell group (fig. S7D), recapitulating the bulk data (27). Notably, we also identify differences among enhancers of the same gene. For example, in the *eve* locus, the stripe 1 enhancer has a much stronger skew for anterior accessibility compared with stripe 2, as has also been previously reported (27). Our single-cell data thus capture the biological variability in enhancer accessibility along the A-P axis, extending previous observations. We similarly could transfer labels from our sci-RNA-seq clusters to spatial coordinates from a spatial enhanced resolution omics sequencing (Stereo-seq)–based spatial study of *Drosophila* embryos at 14 to 16 hours and 16 to 18 hours of development (28). Using the assigned annotations of tissues from the spatial study, we observe a correspondence with our cluster annotations, which again suggests the spatial-relevant variability present in these data (fig. S7E).

### Tracing dynamic gene modules across development

**Fig. 4.**
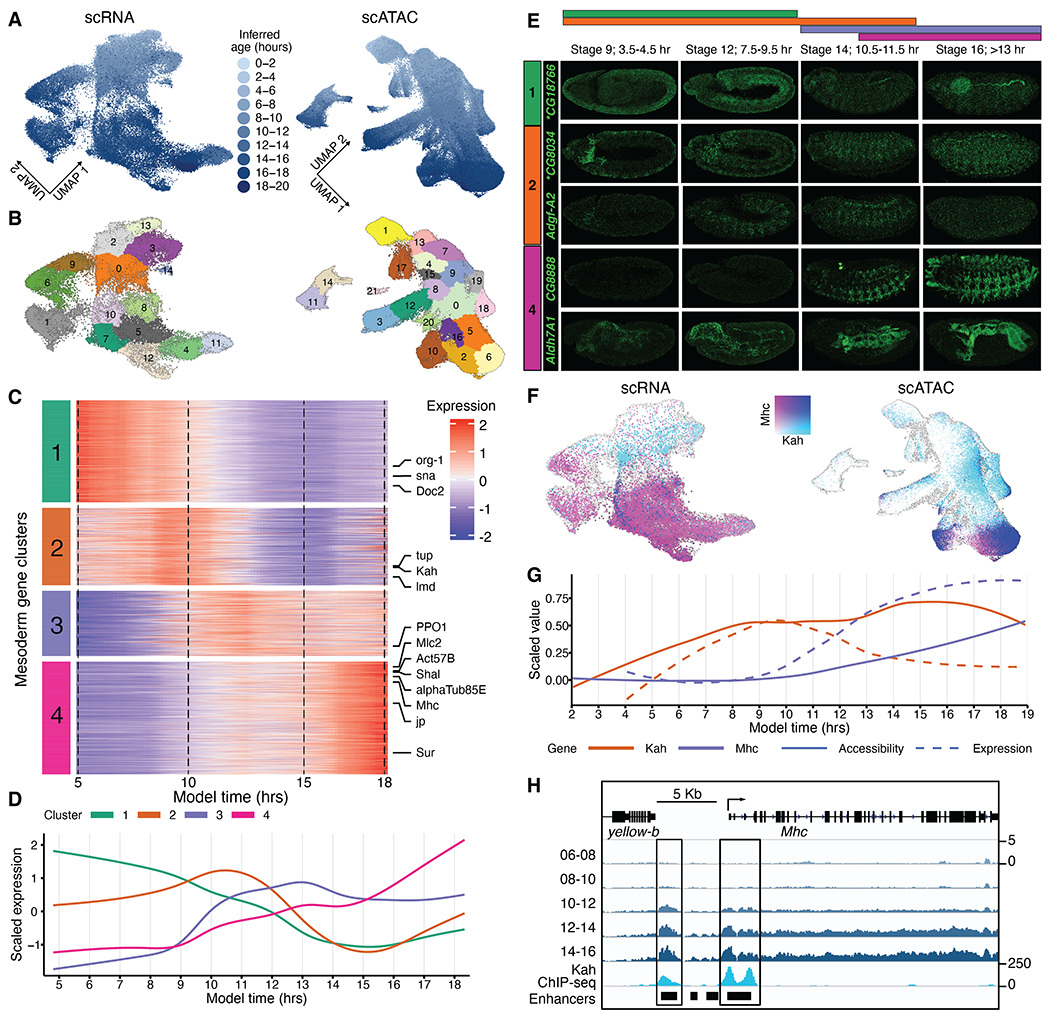
Dynamic regulation of mesoderm-specific gene modules. (**A**) UMAP of scRNA (left) or scATAC (right) data for all mesodermal cells, colored by inferred developmental age. (**B**) Same as (A), but colored as reprocessed leiden-based clusters. (**C**) Normalized expression of mesoderm genes across inferred developmental time. (**D**) Average expression of the gene modules across inferred time. (**E**) In situ hybridization experiments validating temporal expression of selected genes with predicted expression in mesoderm and muscle (asterisks indicate see supplementary note 3). (**F**) Same as (A), but expression of *Kah* (cyan) and *Mhc* (purple) is overlaid. Points from cells that express both *Kah* and *Mhc* are colored dark blue. (**G**) Comparison of gene activity score (solid line) and gene expression (dashed line) over the continuum of inferred developmental age for *Kah* (cluster 2) and *Mhc* (cluster 3) in mesoderm-annotated cells. Gene activity scores and expression were binned into 100 equal partitions by inferred age, averaged, and scaled to 0 to 1 with min-max values. (**H**) Chromatin accessibility profile surrounding *Mhc* for pseudobulk mesoderm cells from 6 to 16 hours inferred time in 2-hour increments, along with Kah ChIP-seq generated from 0- to 16-hour whole embryos (14).

To further leverage continuous views of unfolding trajectories, we next explored the gene regulatory modules active in germ layer–specific development. We focused on the mesoderm and its derivatives as a complex, well-characterized system that we and others have studied previously (11, 13, 29, 30). For this, we selected all cells corresponding to mesoderm-derived cell states, collectively 51,338 (scRNA) and 200,907 (scATAC) profiles across 4 to 20 hours and 2 to 20 hours of inferred developmental age, respectively (Fig. 4, A and B).

Focusing first on RNA, we selected the top 2000 most variable genes. After normalizing expression values to be comparable across time, we used dynamic time warp clustering to group genes into four clusters with distinct temporal regulation (Fig. 4C, fig. S8A, and table S6). These clusters define broad successive waves of gene expression during mesoderm development (Fig. 4D) and notably exhibit similarly ordered waves of chromatin accessibility (fig. S8, B and D, and supplementary note 2). Gene pathway enrichment suggests different functional roles for each cluster (fig. S8C). Cluster 1 genes (*n* = 571) are highly expressed from the beginning of mesoderm development (directly after gastrulation; 4 to 9 hours); are enriched for TFs (*P* = 1.4 × 10^−6^); and likely represent a mixture of genes involved in progenitor cells, mesoderm development, and transcriptional activation (Fig. 4D and fig. S8C). Cluster 2 genes (*n* = 433) peak at ~9 to 11 hours, during the subdivision of the mesoderm into different muscle primordia and their subsequent specification. This cluster is enriched for genes involved in mesoderm development, including myoblast fusion and myotube differentiation, while losing enrichment for stem cell and self-renewal terms (Fig. 4D and fig. S8C). By contrast, cluster 3 genes (*n* = 365) initiate expression at ~10 hours and steadily increase to the end of embryogenesis, whereas cluster 4 genes (*n* = 631) only switch on at ~15 hours, during muscle terminal differentiation. The last cluster lacks enrichment for TFs and rather includes genes involved in myofibril assembly and muscle assembly and maintenance as well as essential contractile proteins for differentiated muscle (Fig. 4D and fig. S8C). We validated the spatiotemporal expression of five poorly characterized genes by in situ hybridization, confirming that they are expressed in the mesoderm or muscle at the inferred time window (Fig. 4E).

The temporal and cell type–specific nature of these expression signatures for both the downstream effector molecules and their upstream regulators should provide the resolution to order genes into putative regulatory hierarchies. For example, several genes with essential roles in muscle differentiation, such as myosin heavy chain (*Mhc*), are present in clusters 3 and 4. Mhc protein plays a critical role in providing muscle-contractile force. Our scRNA data show increasing *Mhc* expression along the muscle lineages in cells with later embryonic ages (Fig. 4, A and F), matching the expression pattern of *Mhc.* Concomitantly, there is a gradual increase in open chromatin at characterized *Mhc* enhancers at later stages along multiple muscle trajectories (Fig. 4G).

Before the expression of *Mhc* and other muscle differentiation genes, we observe transient expression of mesoderm-associated TFs (cluster 2; Fig. 4C). One example is *Kahuli* (*Kah*), a TF associated with muscle development, which has peak expression at 10 hours of embryogenesis (cluster 2; Fig. 4, C, D, F, and G). To investigate the relationship between open chromatin and gene expression, we computed gene activity scores, defined as the sum of sci-ATAC-seq reads in the gene body and the 2 kb flanking the transcription start site (TSS). The gene activity scores for both *Mhc* and *Kah* recapitulate their sequential temporal patterns of expression, with *Kah*’s activity signature appearing earlier along the mesodermal trajectories compared with that of *Mhc* (Fig. 4, F and G). To determine the extent to which we could map the exact ordering of accessibility and expression changes, we overlaid the scaled expression values and gene activity scores averaged across bins with equal numbers of cells (Fig. 4G). Notably for *Kah,* gene expression temporally follows the trajectory of the corresponding gene activity score based on open chromatin, suggesting an ordering where first the gene body becomes accessible followed by accumulating levels of the corresponding transcript; however, this was not the case for *Mhc,* for which expression and accessibility increased in tandem (Fig. 4G). Kah binds to several characterized *Mhc* enhancers near the gene’s promoter, as observed in bulk ChIP sequencing (ChIP-seq) data (14), which suggests a regulatory link between Kah and *Mhc* expression (Fig. 4H).

To extend this analysis more globally, we searched for TF motifs enriched in putative enhancers (mesoderm-specific scATAC peaks 1 to 10 kb upstream of the TSS) of genes belonging to each of the four scRNA mesoderm expression clusters. This identified 458 TF motif-to-cluster enrichments (*q* < 1 × 10^−3^ and presence in >1% of target peaks; table S7) corresponding with 152 unique TFs. Of these, 31 are TFs whose expression changes along mesoderm differentiation and are thus included in the expression-based clustering (table S7). These 31 include many TFs essential for mesoderm development, including a number of direct target genes of the master regulator Twist (the functional ortholog of MyoD) at the beginning of mesoderm development (e.g., *hb, en, Ubx,* and *pb*), and concordantly expressed in the first temporal cluster. These factors have many functions, including setting up the segmentation of the mesoderm, regulating the expression of somatic muscle identity genes, establishing midgut constrictions in the visceral mesoderm, and heart patterning. Other examples from the second and third temporal clusters are genes required for cell fate specification of somatic muscle founder cells (e.g., *Six4* and *ap*) and heart development (e.g., *tup* and *Lim3*).

We note that this approach may miss the contribution of important TFs that were not variably expressed in mesoderm. In particular, if a TF is variably expressed and has corresponding variability in motif activity, this TF is likely active. However, this does not imply that all expressed TFs are active (e.g., there may be coactivators or posttranslational modifications that are required). This caveat notwithstanding, these analyses highlight the potential for further discovery of coregulated gene modules related to distinct germ layers or cell types.

### Nominating stage– and cell type–specific TF regulators

We next investigated whether we could leverage the diversity of cell states across embryogenesis to infer which TFs drive specific programs of cell type differentiation. For this, we used all scATAC clusters at all time points (in contrast to the scRNA-focused cluster analysis above) and searched for differential enrichment of TF position weight matrices (PWMs) within each cluster’s open chromatin regions.

**Fig. 5.**
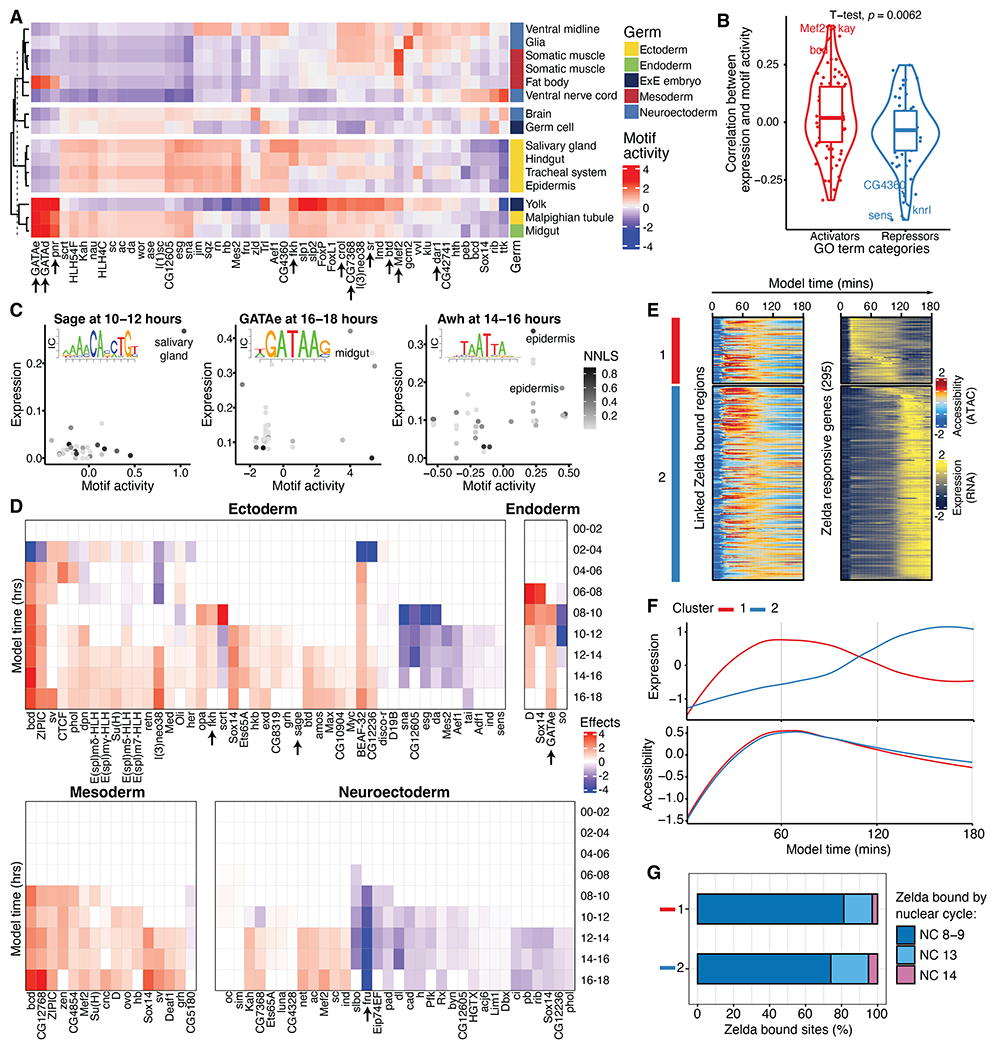
Integration of scRNA and scATAC data to identify TFs with potential regulatory roles across differentiating tissues and developmental time. (**A**) Heatmap with averaged chromatin accessibility differences associated with the 50 most variable TF-specific motifs from all cells in annotated ATAC-seq clusters from 10 to 12 hours. Arrows indicate TFs discussed in the main text. (**B**) Correlation between expression and motif-associated accessibility grouped by expression activation- or repression-associated GO categories. TFs in GO pathways for gene activation are linked to increasing chromatin accessibility. (**C**) omparison of gene expression (*y* axis) and motif-associated chromatin accessibility (*x* axis) across NNLS-linked clusters for the TFs Sage (left), GATAe (middle), and Awh (right). Each TF’s corresponding PWM is inset in each plot, with the size of each base scaled by information content. (**D**) Heatmaps of estimated effects of gene expression at predicting motif-associated chromatin accessibility changes through time in different germ layers. Displayed TFs had three or more consecutive time windows with a significant (*P* < 1 × 10^−3^) and sign-consistent effect. Arrows indicate TFs discussed in the main text. (**E**) Heatmap of expression at Zelda-responsive genes (right) and aggregated chromatin accessibility (left) at their Zelda-bound cis-regulatory regions (38, 39). Values were averaged in 1-min windows over 0 to 3 hours of development. The red and blue bars to the left indicate two temporal clusters of expression of Zelda-responsive genes. (**F**) Smoothed average expression and accessibility for the two Zelda temporal clusters from (E). (**G**) Proportion of accessible regions from (E) that are bound by Zelda in clusters 1 and 2 in ChIP-seq data (39) from different nuclear cycles (NCs).

We first characterized enrichments across clusters from the 10- to 12-hour time window based on predicted time (Fig. 5A). Encouragingly, hierarchical clustering of the enrichment profiles of all associated PWMs grouped each cluster roughly by germ layer (this was also observed in other time windows; fig. S9A). The nonmyogenic mesoderm (fat body) and myogenic mesoderm (somatic muscle) cluster together (Fig. 5A). Open chromatin regions in the myogenic clusters are enriched in motifs for many TFs known to play a role in muscle development, including Mef2 and Fork head (Fkh) TFs. The myogenic clusters also appear close to two neuronal clusters (Fig. 5A), which is driven by shared motif enrichment with neuroectoderm and glial cells, particularly many C2H2 zinc finger TFs, including Btd, CG7368, Crol, Sr, and Dar1. Many of these factors have known roles in neuronal development (e.g., Dar1), whereas Stripe (Sr) is essential for muscle tendon cell fate and muscle attachment in the epidermis at late stages of embryogenesis (31).

Because members of the same family of TFs typically recognize similar motif sequences (e.g., GATAe, GATAd, and pnr), it is often difficult from motif analysis alone to pinpoint the responsible TF. To address this, we leveraged our scRNA data to identify the most likely active TF on the basis of its expression within the clusters among all factors that share the same motif binding pattern. First, we used a regression-based framework to integrate the scATAC and scRNA datasets and identify links between the different cell clusters (1, 6). Specifically, we adopted a nonnegative least square (NNLS) matrix factorization approach that decomposes expression data as a mixture of components derived from proximal gene activity scores generated from the scATAC data. Despite possible temporal differences between accessibility and expression, NNLS identifies stronger links between clusters from the same 2-hour window compared with those from adjacent 2-hour windows (fig. S9B). We also inferred NNLS links in the opposite direction by decomposing proximal gene activity scores by gene expression associated with scRNA clusters. For each cluster of a given data type, the result of NNLS factorization is a mixture proportion of clusters from the other data type, where a higher value represents a stronger association between the scRNA and scATAC cluster (fig. S9, C to F, and table S8). This factor decomposition approach resulted in a strong linkage (NNLS-mixture coefficient of >0.1) of 120 cell state clusters present in the same inferred time windows, with most of the strongly linked clusters being from 4 to 6 hours onward. Upon manual inspection, many linked scATAC and scRNA clusters, which had been independently annotated, are from matching tissues. For example, from the 10- to 12-hour window, the epidermis cluster (cluster 0) in scATAC data was matched to the epidermis (cluster 3) in scRNA data. Altogether, of 21 ATAC clusters from the 10- to 12-hour window, 16 had a linked RNA annotation with a NNLS correlation value >0.1, of which 14 were between comparable tissue annotations.

These integrated scRNA and scATAC clusters, which span 0 to 18 hours of embryogenesis, enabled a more direct analysis of the role of specific TFs in different cell types’ differentiation. We reasoned that active TFs should be more highly expressed in cell types for which they have a functional role, and their associated PWM should be more enriched or depleted in accessible regions when the TF is activating or repressing expression (6). In line with this, correlation values between motif-associated accessibility and gene expression were shifted toward more positive values for TFs annotated [by gene ontology (GO)] as activators and toward more negative values for annotated repressors (Fig. 5B and table S9), a trend also observed in human fetal tissues (6). This approach of linking TFs’ cluster-specific expression and motif enrichments allowed us to nominate TFs as active at specific times in specific tissues (Fig. 5C). For example, this analysis predicts a specific role for Sage in salivary gland development, as the salivary gland is the only cell type exhibiting both high expression of the *sage* transcript and high accessibility of the Sage-associated PWM (Fig. 5C, top). This finding matches the essential role for *sage* in salivary gland development, as determined by genetic loss-of-function analysis (32). Similar predictions were made for GATAe in the midgut at 16 to 18 hours and Awh in the epidermis at 14 to 16 hours (Fig. 5C, middle and bottom), matching the functional role for both TFs in midgut endoderm (33) and epidermis (34, 35) development, respectively.

To expand this analysis and systematically nominate TFs that potentially drive germ layer–specific differentiation programs, we fit a linear model that predicts a TF’s motif-associated chromatin changes from an estimated effect of an interaction term that includes the expression level of the TF in a specific germ layer and time window. Our model’s effect estimates can identify TFs with specific motif activity in particular germ layers and suggest time windows from which a TF initiates its activity. For example, the model refined the role of Sage as becoming active in the ectoderm germ layer specifically from 10 to 12 hours onward and the activity of GATAe initiating in the endoderm from 8 to 10 hours onward (Fig. 5D, top). Such a model encompassing germ layers across development time may also identify additional likely coactive TFs. For example, in addition to Sage, we found Fkh to be both coexpressed and coactive in the ectoderm—a TF reported to act together with Sage to activate salivary gland–specific genes (36).

This analysis also generated additional interesting findings for other time points and germ layers [e.g., Fruitless (Fru); supplementary note 4 and Fig. 5D]. Altogether, from eight high-level germ layer–associated tissue annotations and 316 TF motifs tested, we identified 1258 significant (Benjamini-Hochberg–corrected *P* < 1 × 10^−3^) TF-to-tissue relationships having both associated expression and chromatin activity at one or more of the nine time windows assessed. We note that in time windows with fewer clusters, the association effect estimates are susceptible to outliers and should be interpreted with caution. Notwithstanding this caveat, these putative assignments represent an extensive resource for future studies (table S10).

To demonstrate the potential of our approach to discover previously unknown putative roles for TFs, we selected four genes and validated whether they were expressed in the linked germ layer by fluorescent in situ hybridization. Although these genes were inferred to have effects in multiple germ layers, their function in either mesoderm (*CG5953* and *CG11617*) or neuroectodermal tissues (*Ets65A* and *CG12605*) was poorly characterized. We confirmed that these factors are in fact expressed in the tissue and time window predicted by our data (fig. S10), suggesting potential roles for these TFs in mesoderm and neuronal development.

To complement the NNLS, we applied a recently developed tool, FigR (37), to further facilitate gene regulatory network (GRN) reconstruction. Because multi-omic ATAC-RNA data from the same cell are required for this task, we first integrated our two independent assays for all cells from 10 to 12 hours using canonical correlation analysis (CCA), identifying the most likely ATAC-RNA cell pairs using geodesic distance–based pairing (37) within the common CCA space. Using these pairs as input for GRN inference with FigR, we linked ATAC peaks to their target genes based on peak-to-TSS accessibility correlation and then computed TF motif enrichments for the linked regions, which, together with the TF expression-accessibility correlation, allowed us to define hundreds of putative activators and repressors at this embryonic stage (fig. S11A). Ranking the TFs by their regulation score (fig. S11B) nominated many activators and repressors that we also identified in the NNLS analysis above, including *l(3)neo38, Lim3, lola, fkh,* and *fru* (Fig. 5D). Focusing on the targets of the regulatory networks across all cells at 10 to 12 hours, we found a large set of genes that appear to be extensively regulated (209 genes with >10 linked regulatory regions) (fig. S11C). We then used the inferred TF activities to explore the factors acting on these genes and their mode of regulation. For example, *tup,* a TF gene required for heart development, undergoes extensive self-regulation (highest motif-RNA correlation) besides being positively regulated by the pan-muscle TF Mef2 and repressed by Run and Opa (fig. S11D). Another top-ranking gene, *chinmo,* an essential neuronal TF, is activated by other nervous system TFs, such as Lim1 and Onecut, and is negatively regulated by Fru (fig. S11E), which we also identified as a neuroectoderm-specific repressor in our NNLS-based analysis (Fig. 5D and supplementary note 4).

Finally, we sought to exploit the fine-grained resolution of inferred nuclear ages to explore the dynamics of an early pioneer TF, Zelda, in regulating chromatin opening followed by transcription during ZGA. We recovered the expression of a set of genes that are Zelda dependent during ZGA (38) and, for each gene, aggregated accessibility at the linked Zelda-bound regions (39) in intervals of 1 min across 0 to 3 hours of embryogenesis (Fig. 5E). Clustering of gene expression identified two broad temporal clusters—a first group of early genes and a second group whose expression increases later, alter ~1.5 hours of embryogenesis. Notably, although accessibility at the Zelda-bound regions linked to the early cluster seems to mirror the temporal expression, regions linked to the late expression gene cluster gain accessibility much earlier, almost as early as the first cluster, which suggests that Zelda is opening these regions for future activation (Fig. 5F). To verify whether accessibility is reflective of Zelda binding, we retrieved Zelda occupancy by nuclear cycle (39), which confirmed that >70% of regions in both temporal clusters are already occupied by Zelda at nuclear cycle 8 to 9, regardless of the associated gene expression (Fig. 5G). Moreover, we found a partial Clamp TF motif match within the second temporal cluster (and no match for the first cluster of a TF that is also expressed), which corroborates its Zelda-paired role during ZGA (40). These results suggest that Zelda establishes chromatin accessibility at a large set of regulatory regions in the early embryo, independently of future gene expression, in agreement with its well-known role as a pioneer factor. In some cases, Zelda possibly also functions as the activator of gene expression (cluster 1), whereas in others it retains a pioneering role, and the gene’s expression is induced by later TFs (cluster 2).

## Discussion

This continuum of *Drosophila* embryogenesis builds on our previous work generating sci-ATAC-seq from three nonoverlapping time windows of embryogenesis (12) and complements other studies performed on specific tissues (30, 41–46) as well as scRNA from entire embryos at one specific stage (7) or on dissected tissues from adults (47). Despite the growing use of single-cell assays to generate large-scale atlases, characterizing fine-scale dynamics of chromatin accessibility and gene expression across developmental time remains a challenge. The large number of cell types and even greater number of cell states and branch points during embryogenesis requires extensive cell sampling at continuous stages to capture regulatory transitions, especially for rare cell types. This is very difficult if not essentially impossible to obtain in most model organisms.

In this work, sampling embryo collections from overlapping 2- to 4-hour time windows, coupled with NN-based inference of more precise nuclear ages, enabled continuous representation of *Drosophila* embryonic development. Other studies have attempted a similar ordering of embryos by developmental time over a 2-day window of mouse development (4). However, because only dozens rather than thousands of mouse embryos can practically be sampled, reliable inference at the scale of hours or minutes is challenging. Similarly, cell age was inferred in *Caenorhabditis elegans* using an independent time series of bulk RNA-seq from whole embryos (48). However, relying on such whole-embryo bulk data to predict developmental age in single cells risks inaccurate aging of rare or transient cell types, especially for more complex organisms.

Computationally, our NN-based inference of developmental age bears some similarity to the concept of pseudotime. As originally proposed, pseudotime aims to serve as “a quantitative measure of progress through a biological process” (18). Analogously, our inferred developmental age tracks the progression of nuclei through development. However, the advantage of pairing an experimental design including overlapping yet tightly defined time windows with temporal ordering is that we can anchor inferred ages to fixed time points, which can potentially lead to a more accurate representation of developmental age for complex cellular trajectories. Put another way, inferred ages are interpretable as units of absolute time that are synchronized across all tissue trajectories. With such a continuum of cellular states, we can begin to infer cell type trajectories that more closely capture the continuous processes of cellular differentiation unfolding within a complex, developing multicellular organism.

There remain further possible improvements to our experimental framework. The alignment or anchoring to real time could be refined with sampling of more tightly staged windows. Multi-omic methods for characterizing multiple data types from the same nuclei may facilitate a joint model that can link paired gene expression and chromatin accessibility (and other modalities) to developmental age inference. There are cases where technical features of the data can lead to increased uncertainty of model predictions. For example, we found that cells annotated as germ cells, from the first collection time window, or with low read count were associated with greater prediction error (fig. S11F). Moving forward, we suggest caution for interpreting findings solely on the basis of inferred nuclear ages from clusters with these features.

The extensive scATAC data, with deep coverage across almost a million cells, likely captured most regulatory elements active during embryonic development and provides a comprehensive resource of potential enhancers for almost any cell type in the embryo. By contrast, our scRNA data had relatively low unique reads per cell and will likely miss some differentially expressed genes in specific cell types. As a result, some delicate analyses remain challenging. For example, we found transcriptional velocity estimates to be unstable with sparse scRNA data, although this issue was mitigated by constructing metacells before velocity analysis (fig. S11G), which may be useful for pursuing targeted questions. In scATAC data, we were able to distinguish XX versus XY nuclei from the proportion of chrX-mapped reads (fig. S11H); however, this was challenging for the scRNA data, again as a result of data sparsity. These shortcomings are to some degree compensated by the large number of cells profiled, as shown by our ability to recapitulate aspects of previously documented heterogeneity even for highly dynamic or restricted phenomena—e.g., ZGA (Fig. 2E).

Overall, this *Drosophila* embryonic atlas provides broad insights into the orchestration of cellular states during the most dynamic stages in the life cycle of the organism. Our results represent a rich resource for understanding precise time points at which genes become active in distinct tissues as well as how chromatin is remodeled across time. The annotation of cell types within these data is an ongoing process and one that is much more challenging at early and mid-stages of embryogenesis as compared with late time points or in adults with differentiated tissues. A comprehensive annotation of embryonic cell states will require a collective effort from the *Drosophila* community. To support these ongoing efforts, we provide information on expression and peaks from all clusters (Fig. 3, A to D) in addition to all intermediate and raw data for further exploration. Although larval stages remain insufficiently profiled, we hope that these data and methods, together with the recently released large-scale adult atlas (47), bring us closer to the community-wide goal of a multimodal *Drosophila* atlas spanning a continuum from zygote to adulthood.

## Materials and methods summary

A detailed version of the materials and methods is provided in the supplementary materials. In brief, *D. melanogaster* embryos were acquired for each of 11 collection windows, and then each pool of embryos was divided, with each half being extracted and fixed for either sci-RNA-seq3 or sci-ATAC-seq3. Libraries were sequenced deeply, and the resulting reads were mapped to dm6 and then processed with a uniform processing pipeline that included quality control (QC) filters for low read depth or high proportions of reads mapping to the mitochondria or ribosomal genes and extensive doublet removal. Between the two data modalities, we obtained profiles for ~1.5 million nuclei, although unique read depth per nucleus was considerably lower for scRNA than scATAC data.

Using the center hour of the collection window, we used several machine learning approaches to fit a model that could infer the age of a nucleus with either gene expression or chromatin accessibility information. Both LL regression and neural networks were fitted to the same training data, with a held-out subset used for model validation and comparison. Given its consistently superior performance, we then relied on specific parameterizations of NN model–inferred ages to reposition nuclei in time. To zoom into fine-scale time points, we binned data by small increments to explore the regulatory dynamics of ZGA. Then, using 2-hour adjacent windows of cells, we computed clusters of similar cells and performed extensive manual review to annotate each cluster’s likely germ layer and cell type. We then used an iterative approach for constructing an acyclic tree of differentiation by identifying the likely precursor cluster for each cluster in a given time window.

Neuroectoderm was iteratively analyzed for deeper annotation of neuronal subtypes, whereas mesoderm was picked for analyses focused on identifying coregulated genes and accessible regions, which were then subjected to ontology and TF motif enrichment analysis. To connect scATAC cell clusters with scRNA cell clusters, we used a regression-based approach (NNLS). Such connections between ATAC and RNA clusters enabled a series of analyses, such as correlating expression with motif accessibility, applying GRN analysis pipelines, etc.

Several additional analyses were performed. We used probabilistic label transfer to map likely cluster annotations from these data to spatial information from patterned DNA nanoballs. We also found it is possible to infer the sex of cells from the proportion of chrX-mapped scATAC reads using a Gaussian mixture model to classify cells. Although RNA velocity was challenging to apply to sparse scRNA data, it yielded more sensible results when subsets of cells were first aggregated to metacells.

The expressions of several genes were verified by fluorescent in situ hybridization: specific neuronal genes active in identified clusters, unexpected coactivity of the *elav* with *binou,* genes active at specific mesoderm time points, and putative active TFs with less-characterized roles in tissue development.

Raw data are available through the Gene Expression Omnibus (GEO). Additional scripts and intermediate files, including bigwigs and a custom web app to visualize UMAPs, are available through our data-sharing website.

## Supplementary Material

Supplementary materials

Supplementary tables

## Data Availability

All other data are in the main paper or the supplementary materials. All raw data are available through the GEO series GSE190149. Additional scripts and intermediate files, including bigwigs for all time windows and clusters, and a custom web app to visualize UMAPs are available at https://shendure-web.gs.washington.edu/content/members/DEAP_website/public/. We downloaded the Kah ChIP-seq data from the ENCODE portal with identifier ENCSR161YRO.
