# Supplementary material for: The continuum of Drosophila embryonic development at single-cell resolution
Source: Science. Author manuscript; Available in PMC 2022 Aug 11. (PMC9371440; doi:10.1126/science.abn5800)
Supplement: Supplementary materials [file NIHMS1828489-supplement-Supplementary_materials.pdf]

Science AAAS

Supplementary Materials for

The continuum of *Drosophila* embryonic development at single-cell resolution

Diego Calderon *et al*.

Corresponding authors: Eileen E. M. Furlong, furlong@embl.de; Jay Shendure, shendure@uw.edu

Science **377**, eabn5800 (2022)
DOI: 10.1126/science.abn5800

The PDF file includes:

Materials and Methods
Supplementary Text
Figs. S1 to S11
References

Other Supplementary Material for this manuscript includes the following:

Tables S1 to S13
MDAR Reproducibility Checklist

Materials and Methods

<u>*Drosophila* embryo collections</u>

Drosophila melanogaster wild-type embryos from Canton S flies were collected as previously described (*12*, *50*). Briefly, embryos were collected on apple-agar plates in two-hour or four-hour windows, following three one-hour pre-lays to clear the females and synchronize the collections. After aging the embryos at 25°C to the specified time window (**Fig. 1A**), the embryos were collected from the plates, cleaned with water and dechorionated in 50% bleach for 2 min. Collected embryos for sci-ATAC-seq were fixed immediately, while embryos for sci-RNA-seq were flash-frozen and stored at -80°C until nuclei isolation. Embryo illustrations adapted from (*51*).

<u>Nuclei isolation and fixation for sci-RNA-seq</u>

Embryos were dissociated in an ice-cold hypotonic lysis buffer (20mM Hepes pH 7.5, 10mM KCl, 1.5mM MgCl2, 1% Superase IN RNAse inhibitor) with a Dounce homogenizer, then incubated on ice for 3 minutes, and filtered through a 40uM cell strainer into a tube that contains 2ml of 1.5 mg/ml DSP or DSS cross linker (Thermo Fisher Scientific). Homogenizer and pestle were washed with an additional 0.5 ml hypotonic buffer, to increase nuclei yield, and the buffer filtered through the cell strainer and added to the cross-linking reaction. Nuclei were cross-linked on ice for 30 minutes, with gentle mixing every 5 minutes. After 30 minutes, 9 ml ice-cold methanol was added (to a final concentration of 80% methanol), and nuclei were further incubated on ice for 15 minutes. Nuclei were rehydrated with 15 ml nuclei purification buffer (NPB) (10mM Hepes pH 7.5, 90mM KCl, 40mM NaCl, 200 ng/ml BSA, 1% SuperaseIN), by adding the buffer drop-wise while gently mixing. Nuclei were collected by centrifugation, and separated from debris by sucrose cushion centrifugation (0.3M sucrose-PBS-triton over 1.4M sucrose-PBS-triton 2000 x g 20 min 4°C). Isolated nuclei were resuspended in 1ml of NPB + 50% glycerol, flash frozen and kept at -80°C until use.

<u>Embryo fixation and nuclei isolation for sci-ATAC-seq</u>

Embryos for sci-ATAC-seq were formaldehyde fixed in cross-linking solution (50 mM Hepes, 1 mM EDTA, 0.5 mM EGTA, 100 mM NaCl, pH 8, 1.8% formaldehyde v/v) with a heptane layer for 15 min with shaking at room temperature. Formaldehyde was quenched with PBS/glycine/triton solution (125 mM glycine + 0.1% Triton in PBS), after which the embryos were washed once in ice-cold PBS, dried and stored at −80 °C in ~1 gram aliquots until further use. Embryo dissociation and nuclei isolation were performed as previously described, using a dounce homogenizer and a 22G needle (steps 1-10) (*52*). Nuclei were then pelleted at 2,000g at 4°C, resuspended in nuclear freezing buffer (50 mM Tris at pH 8.0, 25% glycerol, 5 mM Mg(OAc)2, 0.1 mM EDTA, 5 mM DTT, 1× protease inhibitor cocktail (Roche), 1:2,500 superasin (Ambion)) by trituration and flash frozen in liquid nitrogen.

<u>*In situ* hybridization experiments.</u>

Whole-mount Drosophila embryo fluorescent *in situ* hybridization (FISH) was performed as previously described (*53*). Briefly, overnight embryo collections spanning all stages of embryogenesis were fixed in 4% formaldehyde (Polysciences #18814-10) for 20 minutes, stained by double fluorescent in situ hybridization, mounted in ProLong Gold Antifade reagent (Thermo Fisher Scientific # P36931) and imaged with a Zeiss LSM780 confocal microscope using a Plan Apochromat 20x/0.8 objective. Images were then visualized in Fiji (*54*).

Sci-RNA-seq methods

sci-RNA-seq3 library construction and sequencing

We performed sci-RNA-seq3 as previously described (1). Several experiments were done and each one included samples from multiple time windows. Associated time-windows for nuclei batches were tracked through specific wells. Additionally, for a subset of the data we included mouse nuclei to serve as a control to determine the rate of cell doublets.

Read processing, nuclei filtering, and doublet removal

The read alignment and gene count matrix generation was performed as previously described (1, *55*). With the single-cell gene count matrix cells with fewer than 250 UMIs, more than 10,000 UMIs, reads mapping to more than 7000 genes, or more than 10% of read counts mapping to ribosomal genes were excluded. Each nuclei was mapped to its original time window from which it was extracted by using the RT barcode.

We performed standard processing of the data split by experiments as recommended by Seurat v3 (*56*) documentation including NormalizeData, FindVariableFeatures (with method set to 'vst'), ScaleData, RunPCA, RunUMAP (with dims set to 1:50 and n.components to 2), FindNeighbors (with reduction set to UMAP and dims set to 1:2), FindClusters. For detection and removal of doublet nuclei we relied on an iterative strategy described in (1). We used a modified version of DoubletFinder (*57*) that could handle large data sets to document for each cell the proportion of neighbors that were simulated doublets. This version uses the first 30 principal components, along with pN of 0.2 and pK of 0.005. The number of nuclei classified as doublets was based on estimates of the count of doublets (barnyard estimate multiplied by recovered nuclei) and were chosen to be the top rank nuclei with the greatest proportion of nuclei that were simulated doublets. Unfortunately, this process alone did not remove all putative doublets that may have been lost among different clusters of cells. Therefore, following clustering of the global dataset, we individually processed each cluster identified and sub-clustered the data. We then eliminated subclusters that were at least 15% classified as doublets. Following removal of all the initial nuclei identified as doublets and subclusters with a large proportion of doublets, we reprocessed the global dataset.

Dimensionality reduction, clustering, and identifying cluster-specific marker genes

The standard seurat processing pipeline, as described in the previous section, was performed on each non-overlapping inferred age window separately. We found that the default Seurat clustering resolution parameter did not capture the dynamics of the presence of different cell types across development. So for each time window we clustered the data with a variety of resolutions (from 0.1 to 1.5 in increments of 0.3) and then computed the within cluster sum of squares (WSS). Visualizing WSS across increasing resolutions we chose the resolution in which there was a visible plateau in the decrease of WSS. The selected set of resolutions are listed in **Table S11**. Finally we clustered the data for each inferred age time window using the per-window choice of resolution parameters. From these clusters we used Seurat's FindMarkers to iteratively loop through all clusters and identify marker genes (with only.pos=T) which were used for cluster annotation. For the set of marker genes used for lineage annotation we excluded genes with 'log.FC < 0.25' or 'min.pct < 25'.

Annotating cell types and tissues

For cluster annotation, we used the Berkeley Drosophila Genome Project (BDGP) database, which includes gene expression patterns of approximately 8600 genes in drosophila staged embryos as detected by *in situ* hybridization (*20*, *21*, *58*). The BDGP database gives a stage-specific expression pattern ("term") for each tested gene during embryogenesis. We used Fisher's test to look for enrichment of BDGP gene expression terms in each cluster's marker genes. Top ten terms per cluster were examined. To pick a specific term out of the top ten, we further examined the BDGP terms of the top 20 marker genes for each cluster.

Clustering genes

Inferred-time associated co-regulated genes specific to certain germ layers were determined with an unsupervised clustering approach. First, we subset the full seurat object that included cells from all time windows to only those cells annotated as a specific germ layer. For example, in analyses shown in **Fig. 4**, we focused on mesoderm. As described previously in the standard seurat processing pipeline, we scaled gene expression values and subsetted to the 5000 most highly variable genes. We constructed 100 bins of roughly equal numbers of cells across inferred time, and then smoothed expression values by computing the average expression of each gene in each window, after trimming 10% of the outlier genes, along with all expression values for cells in time proximal bins from a sliding window across inferred time. Once again we subsetted the genes to the top 2,000 that were highly variable across these smoothed time windows and then these variable expressed genes were scaled and centered to have mean=0 and sd=1. Finally, we performed dynamic time warp clustering with the 'tsclust' function from dtwclust v5.5.10 with type='partitional', distance='dtw_basic', and centroid='pam' on these scaled gene values to identify co-regulated genes (*59*). To choose the number of clusters (k) we performed clustering with all k values from 2 through 35 and then selected the earliest k at which several metrics of clustering metrics began to plateau (**Fig. S8**). We downloaded the Kah ChIP-seq data from the ENCODE portal (*60*) with the following identifier: ENCSR161YRO.

Gene pathway enrichment analysis

We were interested in relating various gene sets to known biological processes. For this task we used FlyEnrichr to perform gene set enrichment analyses (*61*, *62*). Gene sets were uploaded to the FlyEnrichr server with the enrichR version 3.0 package for R available on CRAN. We restricted our results to enrichments in the "GO Biological Process 2018" database and the "RNAi Screens from GenomeRNAi 2017" database.

RNA velocity analysis

We randomly sampled 20,000 cells from 3 adjacent time windows (10-12 hr, 12-14 hr, and 14-16 hr) and then randomly grouped cells into 100 meta cell bins and aggregated all reads per meta cell bin. We then used the scVelo software package version 0.2.2 (*63*) and the standard processing pipeline to estimate the RNA velocity graph, which we then visualized as a directional vector in the first two principal components space, thus preserving the linear interpretability. The script for performing this analysis is included in our data sharing page.

Sci-ATAC-seq methods

sci-ATAC-seq3 library construction and sequencing

To create the sci-ATAC-seq3 libraries, we followed the protocol from (6). As previously described, frozen fixed nuclei were thawed, re-permeabilized in Omni lysis buffer (*64*), and diluted in ATAC–resuspension buffer (RSB) buffer (10 mM Tris-HCl pH 7.4, 10 mM NaCl, 3 mM MaCl2) supplemented with 0.1% Tween-20. We profiled 11 samples corresponding to developmental time windows in 2 experiments and one barnyard sample per experiment. For each time window, 50,000 cells were deposited across wells of a LoBind 96-well plate, 8 time windows per experiment (across 11 wells) in addition to a barnyard sample (across 8 wells) which is a mixture of mouse CH12-LX and human GM12878 cell lines. Re-permeabilized nuclei were tagmented with Tn5 enzyme at 55°C for 30 min then stop reaction buffer (40 mM EDTA with 1 mM Spermidine) were added afterwards and incubated at 37°C for 15 min. Pooled tagmented nuclei from each time window were pooled, pelleted and washed. Phosphorylation master mix [1X polynucleotide kinase (PNK) buffer, 1 mM rATP, T4 PNK] was added to the washed tagmented nuclei and incubated at 37°C for 30 min. Next, ligation master mix [1X T7 ligase buffer, N5_splint, T7 DNA ligase enzyme] was added directly to the phosphorylation reaction followed by 384 distinct N5_oligos then incubated at 25°C for 1 hour. Stop reaction mixture was added to the ligation reaction and incubated at 37°C for 15 min. All wells were pooled then transferred into a 50-ml falcon tube, pelleted and washed with ATAC-RSB with 0.1% Tween-20. N7 ligation master mix [1X T7 ligase buffer, N7_splint, T7 DNA ligase] were added to the washed pellet and aliquoted into four 96-well LoBind plates. 384 distinct N7_oligos were added across four plates of N7 ligation and incubated at 25°C for 1 hour, then stop reaction mix were added for another 37°C for 15 min incubation. Afterwards, all wells were pooled and transferred to a clean 50-ml falcon tube then washed in ATAC-RSB with 0.1% Tween-20 before resuspending in Qiagen EB buffer. The ligated and washed nuclei were counted and aliquoted at 1000-3000 nuclei per well across four 96-well LoBind plates. Proteinase K and 1% SDS were added to the nuclei to reverse crosslink and incubated at 65°C for 16 hours. To determine the optimal cycle number, a test amplification was performed and monitored with SYBR green on a handful of wells of a reversed crosslink plate. The remaining plates were processed on the basis of the test PCR result. PCR amplifications were performed using NEBNext High Fidelity 2X PCR Master Mix, BSA [bovine serum albumin], indexed P5 oligo, and indexed P7 oligo. All wells were pooled and purified with Zymo Clean & Concentrate-5 and further purified with 1X AMPure bead to get rid of any remaining primers and adapter dimer. Purified libraries were quantified on an Agilent 4200 Tapestation System using D5000 reagents and screentape. Libraries were then diluted to 2 nM for sequencing using a custom recipe and primers on a NextSeq 500 to assess library complexity then further sequenced on an Illumina NovaSeq 6000 sequencer with custom sequencing recipe and primers.

Data processing for sci-ATAC-seq3

The sci-ATAC-seq3 raw reads were processed using the pipeline described in (6). Reads were mapped to the dm6 reference genome. The non-duplicate fragments are used for peak calling with MACS2 (*65*) in each sample, and then merged together with bedtools (*66*). We generate sparse matrices counting reads falling into each 5 kb window in the genome for cells passing a sample-specific threshold for each sample. We also generated sparse matrices counting reads falling into the merged peak set and into gene bodies plus 2kb upstream regions (proximal gene activity matrices). The barnyard sample was mapped to the merged hg19-mm9 reference genome to estimate the collision (two cells receiving the same barcode by chance) rate in the experiment.

Dimensionality reduction and clustering

The downstream analysis steps also closely follow the pipeline described in (6). We binarized the window-by-cell matrices for downstream analysis. We merge the binary matrices for time windows profiled in both experiments. We exclude peaks on sex chromosomes and peaks accessible in less than 1% of cells. We use latent semantic indexing with log-scaled term frequency to normalize the binary matrices. We use singular value decomposition on the normalized matrices to generate principal components (PCs). Retaining the 2nd through 50th PCs (discarding the 1st PC, which is generally correlated with read depth) and applying L2-normalization on the PCA matrix, we generate a low-dimensional representation for each time window. The normalized PCA matrices are used for Louvain clustering and UMAP (min.dist=0.3) as implemented in Seurat v3 (*56*). For the Louvain clustering, we used resolution of 0.3 for the first round clustering and we varied the resolution parameter per time window for the final clustering after cluster specific peak calling. To select the clustering resolution parameter for the scATAC-seq clustering in each time window, we computed clusters with various clustering resolutions and selected the one at which the proportion of variance explained by the clustering plateaued (elbow method). We did not observe batch effects between the two experiments (**Fig. S2C**) in any developmental time window and thus did not run batch removal algorithms.

Doublet identification

The individual experiments had low estimated rates of doublets (**Fig. S2**). Based on the barnyard samples we expect 1.8% of doublets within our dataset (**Fig. S2**). We use a modified version of the scrublet algorithm (*67*) to calculate a per cell doublet score and set a threshold of the 95th percentile. For each developmental time window, cells with doublet scores above the threshold and clusters with over 25% cells of above the threshold are removed. The remaining cells for each developmental time window are re-embedded and re-clustered using the pipeline described above.

Cluster-specific peak calling

In order to generate a comprehensive set of peaks, we split the fragments files by cells in each Louvain cluster and call cluster-specific peaks with MACS2. The summit of each peak is extended to 150bp and then merged into a master peak set with bedtools. The peak-by-cell matrices are re-counted with the new peak set, and the cells for each developmental time window are re-embedded and re-clustered using the pipeline described above. In total, we identified 110,185 peaks (median length = 217 bp) that collectively cover 22% of the dm6 genome. We compared this peak set with sets of known elements, including annotated TSS sites (extended 2 kb upstream and 200 bp downstream), peaks identified in (*12*), curated set of known embryonic enhancers (Bonn et al. 2012, Kvon et al. 2014, Rivera et al. 2018), and bulk DHS peaks (Reddington et al. 2020). For each pair of sets of elements, we calculate the portion of elements overlapping elements in the other set by 1 bp overlap, and vice versa. The peaks in this study overlap over 85% of each set of the known elements, while the known elements overlap less than 50% of the peaks in this study.

Global embedding

In order to visualize global trends of development based on the regulatory landscape, we randomly sampled 20,000 cells from each 2-hour developmental time window and 40,000 cells

from each 4-hour developmental time window, merged the chromatin accessibility profiles and embedded the cells together using the pipeline described above.

Cell type annotation

To transfer cell type labels from the sci-ATAC-seq dataset in (*12*), we use the integration pipeline implemented in Signac V1 (*68*) on five developmental time windows (2-4, 4-8, 6-10, 8-12, 10-14) that overlap one of the three time windows (2-4, 6-8, 10-12) profiled in (*12*). We collapse the cell type subsets (*e.g.* CNS A, CNS B to CNS) in (*12*) to a set of 37 cell type labels and count the number of cells in our dataset with each cell type label in each cluster. We assigned the most prominent cell type label to each cluster. For most clusters we could unambiguously assign a cell type label. We propagate the cell type labels to all developmental time windows through connections identified in the developmental tree described in the 'Reconstruction of the developmental tree' section. Since our study generated a much larger dataset, we expect to observe cell types that are not identified in (*12*). Therefore, we also refined the transferred labels based on differentially accessible (DA) peaks and differentially expressed (DE) genes. DA peaks are calculated from the binary peak matrix with the FindMarkers() function as implemented in Seurat V3 with test.use='LR' and logfc.threshold=0.1. Peaks with a p-value less than 0.05 from the test are included in the foreground set and the remaining are included in the background set. The two sets of peaks in each sample are overlapped with a previously compiled embryonic enhancer database (CAD; Bonn et al. 2012, Kvon et al. 2014, Rivera et al. 2018) of the closest matching time stage. We use a Fisher's exact test to look for 'terms' in CAD that are enriched in the foreground set compared to the background set. We used terms with adjusted p-value less than 0.05 to refine the transferred labels. DE genes are calculated from the proximal gene activity matrix with the FindMarkers() function as implemented in Seurat V3. We look for 'terms' in the BDGP that are enriched with the same procedure as in sci-RNA-seq annotation.

Motif analysis

We calculate a per-cell motif activity score for known motifs with PWMs in the CisBP database (*69*) using chromVAR (*70*). Within each cluster, chromVAR calculates a bias-corrected deviation of accessibility of each cell from the average accessibility in all the cells. We also use Homer (*71*) to identify motifs enriched in the mesoderm-specific gene regions. We take the peaks open in over 2% of mesoderm cells and that overlap the 1kb-10kb region upstream of the TSS of the four clusters of genes in **Fig. 4C** as the target, peaks open in less than 2% of mesoderm cells and overlapping the 1kb-10kb region upstream of the TSS of non-mesoderm genes as the background, and run *findMotifsGenome* with the parameters *-cpg -size given* (**Table S7**).

Data analysis

Inferring developmental time

To estimate the developmental time stamp for each single cell, we trained lasso linear and neural net-based models using the window-by-cell read counts matrix for sci-ATAC-seq and the expression read count matrix for sci-RNA-seq to predict the midpoint of each developmental time window (*e.g.,* 7 for the 6-8 hours developmental time window). First, we equally subsampled cells for each time window to normalize the number of included cells per hour of collection time (*i.e.* 4 hour windows had twice as many nuclei as two hour windows). Following even subsampling, all genes and peaks that were constant values were removed. Then, we split the equally time

subsampled data into 11 partitions of cells. The first 10 partitions were used for 10-fold cross validation to test many different model parameter choices (outlined below for each model type) and the final held out 11th partition was used as a test data set to evaluate the final models. To be clear, all model fitting described below was performed with the first 10 partitions of cells.

The lasso model was trained with cv.glmnet() as implemented in glmnet (*72*). In this case, the only parameter being fit by cv.glmnet() is the strength of the lasso penalty. We use the trained model to infer the developmental time for all the cells with predict(), setting s='lambda.min'.

We used tensorflow v 2.6.1 (*73*, *74*) to fit all neural net models using a fully connected, feedforward 6-layer (4 hidden) neural network. For the first hidden layer we included variable l1 or l2 regularization with the 'kernel_regularizer' parameter, and in the last layer we optionally constrained the output to within 0-20 with either a sigmoid or tanh activation function. All hidden layers used relu activation functions. The ATAC and RNA model were very similar, and only differed in the number of units per hidden layer. For ATAC there were 10, 100, 60, 20, and for RNA there were 5, 100, 50, 20, 1 units per hidden layer. Moreover, the RNA model input was scaled with a 'Normalization' layer from tensorflow, whereas the ATAC model was not. Finally, we used 'SparseTensor' to encode the ATAC data.

For fitting these models we optionally used either the standard mean squared error from the center hour of the collection window (MSE) or a custom loss function based on MSE except the error is set to 0 if nuclei are placed within the correct collection window. We used 10-fold cross validation to estimate the generalization error of these models with a variety of parameterization choices for the L1 penalty [values=1, 0.1, 0.001, 0.0001, 0.00001, 0.000001, 0], L2 penalty [values=1, 0.1, 0.001, 0.0001, 0.00001, 0.000001, 0], activation function (linear, sigmoid, tanh), and loss (MSE, custom). We selected the two best performing models (one using MSE and the other using the custom loss) based on the best median MSE and the proportion of nuclei placed into the correct collection window across all the 10-folds.

We split the developmental time frame of 0-20 hours into 10 non overlapping 2 hour inferred time windows and reassign cells to each inferred time window based on their inferred times. The cells for each inferred time window are re-embedded and re-clustered using the pipeline described above.

Scripts for arranging the data and fitting the models can be found on our supplementary data sharing website.

Initially, when we tested these time inference models with bulk RNA/ATAC/DNase-seq libraries, the age estimates were outside the hour range of this experiment even though they produced the correct temporal ordering. We suspected that these large inferred time values were due to the differences in the read depth of the bulk libraries versus single-cell nuclei. To address this we simulated subsampling reads the median number of reads per nuclei (377 UMI for RNA; 5294 unique reads for ATAC) with the 'rmultinom' function from base R with the parameter 'prob' set to the read counts of the bulk library. To integrate over sampling error, from each bulk library we repeated the subsampling process 100 times and then averaged over the model-inferred

predictions. This process of adjusting the read distribution of the bulk libraries to match the single-cell nuclei resulted in time predictions that were in the same scale as our time course experiment.

Inferring nuclei sex

To infer whether each nuclei was XX or XY we used the proportion of chrX-mapped sci-ATAC-seq reads as a summary statistic and identified two distinct populations **Fig. S11H**. After filtering out nuclei with 0 chrX-mapped reads or if they were in the tails of the distribution (prop. X > 0.07 and prop. X < 0.22), we fit a gaussian mixture model with the 'normalmixEM' function from the mixtools package version 1.2.0 (*75*) and the parameter 'k'=2. The gaussian mixture model was successfully able to separate these two populations of cells into a set with likely XX genotype (more proportion of chrX-mapped reads) compared to a set of cells that were likely XY genotype (less proportion of chrX-mapped reads). To verify that these classifications were accurate, we could validate that the XY genotyped cells were indeed male cells by verifying that they were enriched for reads mapping to the Y chromosome. Cells with a >95% probability of being classified as XX, based on this mixture model fit, only 8% of these nuclei had 0.05% or more proportion of Y-mapped reads. In contrast, for confidently predicted male cells (*i.e.* cells with >95% probability of being classified as XY), 42% had 0.05% or more Y-mapped reads. The full annotations of nuclei genotype are now included in the supplementary tables. Unfortunately, this analysis was not possible with the sci-RNA-seq data likely due to dosage compensation and too few reads per nuclei.

Reconstruction of the developmental tree

To connect each cell state observed in a predicted time window with its most probable ancestor cell state from the previous predicted time window, we use the k-NN approach described in (*76*). We took cells from neighboring predicted time windows and co-embedded them using the pipeline described above. For cells in the later inferred time window, we identify ten k-NN cells in the previous inferred time window based on 50 PCs. The edge weights connecting the clusters in each inferred time window to clusters in the previous inferred time windows are set to the percent of cells in each cluster that has majority k-NNs from a cluster in the previous inferred time window. Edge weights < 0.2 are removed. Branches with only one leaf and nodes in the 18-20 hours inferred time window are pruned for the developmental tree shown in **Fig. 3C-D**.

Spatial analysis

A tranche of spatial data was recently released including a study on *Drosophila* using SpaTial Enhanced REsolution Omics-sequencing (Stereo-seq) (*29*), which was collected from late-stage embryos and all stages of larvae. The late-stage embryos were from corresponding windows as our embryo samples 14-16 h and 16-18 h after egg laying. This technology is not single-cell based, but instead patterned DNA nanoballs are placed on a slide that then capture RNA transcripts which are then sequenced and associated back to the slide spatial position of the nanoball slide spatial position. With an anchor-based integration *FindTransferAnchors()* and *TransferData()* from Seurat v3 (*56*), we performed probabilistic label transfer to assign our cluster labels to each patterned nanoball's spatial location. Using the assigned annotations of tissues from the original study as reference above, we observe a correspondence with our cluster annotations (**Fig. S7E**).

Connecting cell states in the sci-ATAC-seq dataset to those in the sci-RNA-seq dataset

In order to identify matching cell states in the sci-ATAC-seq and sci-RNA-seq datasets, we implemented the non-negative least squares (NNLS) approach as described in (1, 6). For the sci-RNA-seq dataset, we calculate an aggregate expression vector for each cluster in each time window by summing the log-transformed normalized UMI counts of all cells in that cluster. For the sci-ATAC-seq dataset we take the proximal gene activity matrix and calculate a similar aggregate activity vector for each cluster. Then we apply non-negative least squares (NNLS) regression to predict gene expression in a target cluster in the sci-RNA-seq dataset based on the gene activity of all clusters in the corresponding time window in the sci-ATAC-seq dataset. The resulting β matrix is denoted $\beta_{ra} \in R^{i \times j}$ where i and j are the number of RNA and ATAC clusters respectively. We then repeat the analysis predicting gene activity with gene expression to obtain $\beta_{ar} \in R^{j \times i}$. For each pair of clusters from the two datasets, we calculate a final beta value based on the element-wise multiplication of the two beta value matrices from NNLS: $\beta = 2(\beta_{ra} + 0.001)(\beta_{ar}^{T} + 0.001)$. Similar clusters, based on patterns of gene expression and the sum of ATAC-seq reads around a gene, in the two datasets have higher beta values.

<u>Determining the relationship between TF expression and associated motif accessibility for NNLS-linked clusters</u>

Relying on the NNLS-based links between clusters of ATAC and RNA data, we next set out to determine correlation between TF-associated motif accessibility and TF-associated gene expression. Presumably TFs with strong correlations are active, with positive correlations indicating TFs likely to be activators of gene expression and negative correlations indicating TFs likely to be repressors of gene expression. For each cluster we averaged the expression of each TF across all cells and averaged the associated motif accessibility score (as described previously) across all cells. Next for each RNA cluster we chose the best associated ATAC cluster as determined through NNLS analysis. For comparison we also performed the vice versa association pairing each ATAC cluster with the top associated RNA cluster and the inferred correlations were similar (**Fig. S9G**). We then computed the spearman correlation and Pearson's R correlation between motif accessibility score and gene expression for each TF with a known associated motif. Additionally, we computed these correlations for each gene at each two hour inferred time window. For the analysis visualized in **Fig. 5D** we fit a linear regression model with 'lm()' in R that predicts the TF motif-associated ATAC-seq activity (from chromVAR) from an interaction term including the expression of the related TF, the germ layer of the cluster, and time window. Prior to fitting this model, the TF expression values were scaled and normalized to have 0 mean and an sd of 1 with 'scale()'. Additionally, we weighed the model observations by the NNLS correlation value representing the strength of the link between the ATAC and the RNA cluster. Intuitively, weighting the observations will increase the contribution from clusters that are strongly linked compared to clusters that have a weak link between ATAC and RNA.

<u>Gene regulatory network analysis</u>

To integrate our transcriptome and chromatin accessibility data, we first created subsampled Seurat objects (5,000 cells) for cells at 10-12 hr and log normalized the RNA counts and ATAC gene activities. We identified variable features for each object using Seurat 'SelectIntegrationFeatures' function and used the features to perform Canonical Correlation Analysis (CCA) with Seurat RunCCA function, in order to create a common embedding for our independently-assayed ATAC and RNA modalities. In CCA space, we used FigR 'pairCells' function to identify pairs of ATAC-RNA cells by geodesic distance-based pairing (*38*). Gene

expression and peak accessibility counts from paired cells were used as input for FigR 'runGenePeakcorr' in order to identify significant (p-value < 0.05) peak-gene associations. The 'runGenePeakcorr' function was slightly modified to accept the *Drosophila melanogaster* dm6 genome (Bioconductor library BSgenome.Dmelanogaster.UCSC.dm6) as a valid input. At this point, the DORC scores and the original RNA counts were smoothed with FigR function 'smoothScoresNN', and fed together with the original ATAC peak counts and the significant peak-gene associations into FigR function 'runFigRGRN', for inference of the gene regulatory networks. The 'runFigRGRN' was slightly modified to accept *Drosophila melanogaster* CisBP PWMs as a valid input. For generating the plots presented in **Figure S11**, we ranked TFs by average regulation score using the FigR function 'rankDrivers', while TFs regulating individual genes were retrieved with the function 'plotDrivers'. Domains of regulatory chromatin (DORCs) were identified with FigR function 'dorcJPlot' with 'cutoff' = 10 (at least 10 significantly linked peaks to be called a DORC) (**Fig. S11C**).

Supplementary Text

Supplementary Note 1: Diversification of non-myogenic mesoderm

We examined the diversification of the non-myogenic mesoderm trajectories in more detail. The non-myogenic mesoderm includes the fat body and haemocyte lineages, which in the embryo includes plasmatocytes and crystal cells. Previous genetic studies demonstrated that the fat body develops from the trunk mesoderm (*77*), similar to the somatic muscle, while the embryonic haemocytes originate from the head mesoderm (*78*). In agreement with this, and without any prior knowledge, the scATAC-derived graphs suggest that the fat body shares a developmental origin with somatic muscle, whereas the haemocytes originate from a separate mesodermal trajectory.

To explore these trajectories at finer resolution, we isolated and re-clustered all cells annotated as plasmatocytes, fat body and crystal cells, using scRNA data from 6-18 hrs. This revealed 17 subclusters, with a large group of early cells (right, clusters 1, 4, 13, 6, 7), that diversifies at 8-12 hrs. The early clusters are defined by the expression of several transcription factors (TFs) including Ultrabithorax (*Ubx*), restricted to the earlier stages, and homothorax (*hth*) and Zn finger homeodomain 1 (*zfh1*), expressed from early to late stages in development. All branches express common haemocyte and fat body marker genes such as the collagenase *Col4a1*, the procollagen lysyl hydroxylase *Plod* and the extracellular matrix gene *Tig*. These branches correspond to sub-trajectories for plasmatocytes (clusters 8, 9, 12, 14), fat body (clusters 0, 3, 10, 11, 16), and additional two muscle clusters (cluster 2, 5). Finally, the crystal cells (a relatively rare cell type, expressing the marker genes *Lozenge*, *PPO1*, *PPO2)* are clearly separated and disconnected from the rest (cluster 15), suggesting that they may have a different developmental origin or, alternatively, that they deviate from a common precursor population earlier than 6 hrs.

Plasmatocytes represent ~90% of the embryonic haemocytes, and are phagocytic cells involved in clearing apoptotic cells (*79*), which requires the expression of the scavenger receptors *crq* and *drpr* (*80*, *81*). We observe both genes, in addition to other scavenger receptors (*e.g. Nimrod C4* and NimB4, specific to later embryonic stages), expressed along the plasmatocyte sub-trajectory. The second function of plasmatocytes is the secretion of extracellular matrix (ECM) proteins, which is also evident from our single cell trajectory revealing dynamic expression of a large repertoire of ECM proteins, such as Papilin (*Ppn*), Peroxidasin (*Pxn*), Glutactin (*Glt*), Tiggrin (*Tig*), basement membrane-associated SPARC, laminin A (*lanA*), and two collagenase IV

molecules - *Col4a1* and Viking (*vkg*). The second major branch is the fat body, which is thought to have similar roles to the mammalian liver and adipose tissues, in addition to having an essential role in immune responses, as the main source of antimicrobial peptides (AMPs) (*82*). We identified 289 genes significantly associated with specific subclusters that highlight the dynamic differentiation of the fat body. For example, the expression of genes involved in lipid transport (*e.g. apolpp* and *apoltp* (*83*, *84*)), increases with the maturation of the fat body cells.

Supplementary Note 2: Tracing dynamic gene modules across development

The analysis steps associated with mesoderm gene clustering of **Fig. 4** were motivated to identify the TFs driving mesoderm-specific expression variability and determine whether we were capable of ordering putative regulatory events (*e.g.* Kah/Mhc). Of course, there are different directions to approach this or related questions. Therefore, we performed a complementary analysis by clustering accessible regions variable in the mesoderm tissue (**Fig. S8D**). The clustering approach was the same as in **Fig. 4** but using variable chromatin accessibility regions from the set of mesoderm annotated nuclei. This analysis identified a similar set of 4 clusters of accessibility elements that are likely involved with regulatory patterning linked to mesoderm differentiation. Additionally, we performed a motif enrichment analysis on these peaks and found 483 significant associations between a peak group and TF (q-value $< 1 \times 10^{-3}$ and a match present in at least 1% of target peaks) from 192 unique TFs. Tying this back to the mesoderm expression analysis, we found that 40 of these TFs were in the set of genes that were variably expressed in the mesoderm tissue. These significant TF associations and the clustered peaks are available from our supplementary data website and **Table S12-S13**.

Supplementary Note 3: Clarification of selected in situ genes

The five genes that we selected for follow-up in situ validation were based on results from our initial nuclei age inference model that used lasso regression to order cells. However, the two genes indicated with an asterisk in **Fig. 4E** (CG18766, CG8034) were missing from the mesoderm gene clustering analysis based on an updated nuclei age model that used deep learning as they were not present in the initial stringent mesoderm subset based on the top 5k variable genes. Upon manual inclusion of these two missing genes into the set of 5k variable genes (so now 5,002 genes), the genes were highly variable following normalization and averaging across time windows (they were included in the second set of 2k variable genes) and were grouped into the correct temporal cluster. Therefore, we have included these genes in **Fig. 4E** grouped by the original mesoderm clustering (based on the initial lasso-based age inference model).

Supplementary Note 4: Nomination of fruitless as a repressive regulator in neuroectoderm

In the neuroectoderm, Fruitless (Fru) is associated with decreasing chromatin accessibility; *i.e.* increasing *fru* expression led to chromatin regions containing the Fru motif to become less accessible (**Fig. 5D**, bottom). Intriguingly, Fruitless is associated with specifying the molecular determinants of male courtship behavior by 'masculinizing' specific neurons (*85*–*87*). However, only recently have studies shown that Fru likely acts through the repression of chromatin accessibility at specific regulatory elements (*88*–*90*).

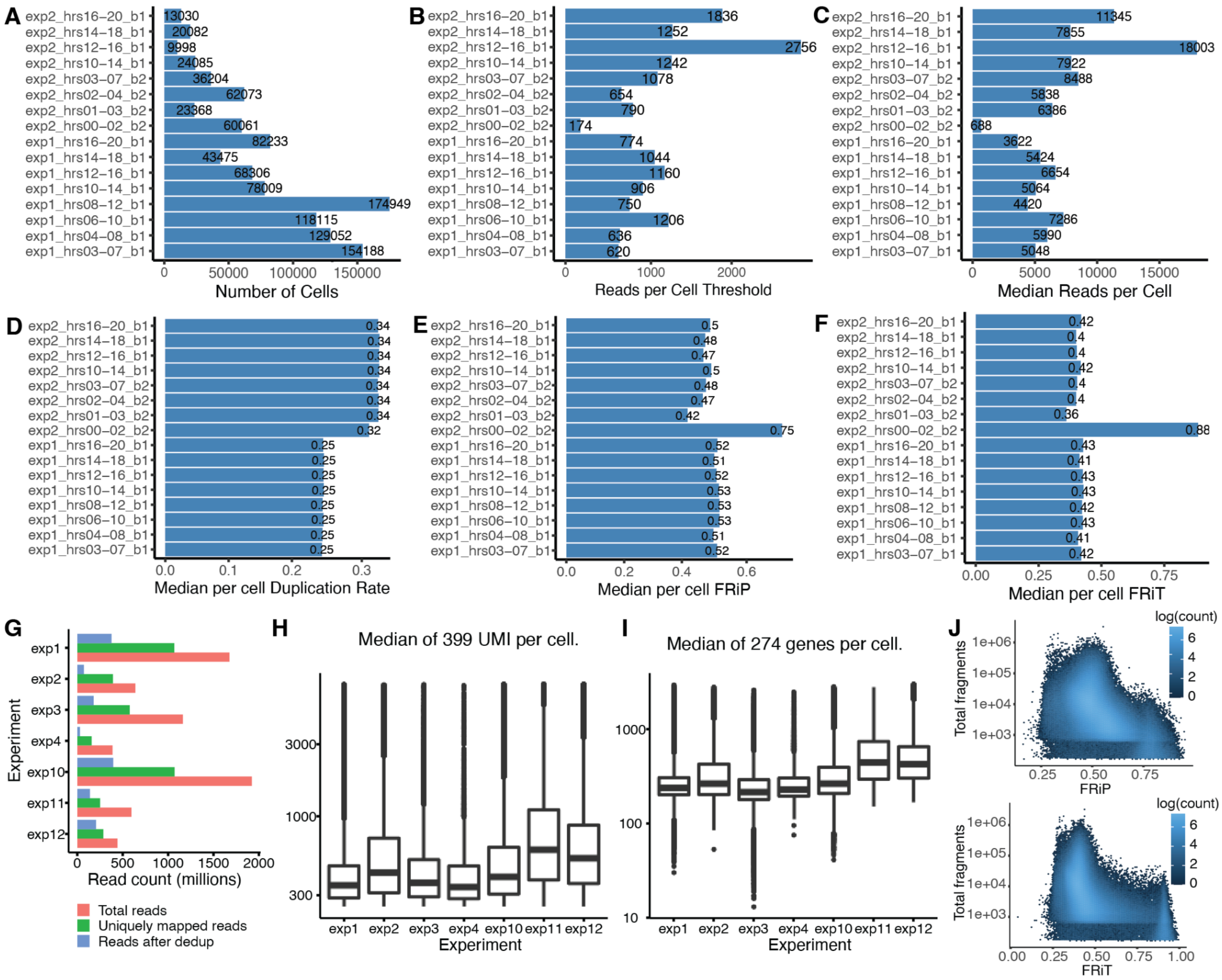

Fig. S1.

QC/stats of the sci-ATAC-seq and sci-RNA-seq datasets. (**A**) Barplot showing number of cells passing threshold in each sample in the sci-ATAC-seq dataset. Samples are labeled by the experiment (exp1/2), developmental hours of the nuclei collection, and the batch in which the nuclei were collected in (b1/2). (**B**) Barplot showing reads per cell threshold for each sample in the sci-ATAC-seq dataset. (**C**) Barplot showing the median reads per cell for each sample in the sci-ATAC-seq dataset. (**D**) Barplot showing median duplication rate per cell in each sample in the sci-ATAC-seq dataset. (**E**) Barplot showing median fraction of reads in peaks (FRiP) per cell in each sample in the sci-ATAC-seq dataset. (**F**) Barplot showing median fraction of reads in TSS sites (FRiT) per cell in each sample in the sci-ATAC-seq dataset. (**G**) Barplot showing the total number of reads, uniquely mapped reads and reads after deduplication in each experiment in the sci-RNA-seq dataset. (**H**) Boxplot showing the number of UMIs per cell in each experiment in the sci-RNA-seq dataset. (**I**) Boxplot showing the number of genes per cell in each experiment in the sci-RNA-seq dataset. (**J**) Hex bin plot showing distribution of nuclei FRiP (top) or FRiT (bottom) scores by total unique fragments.

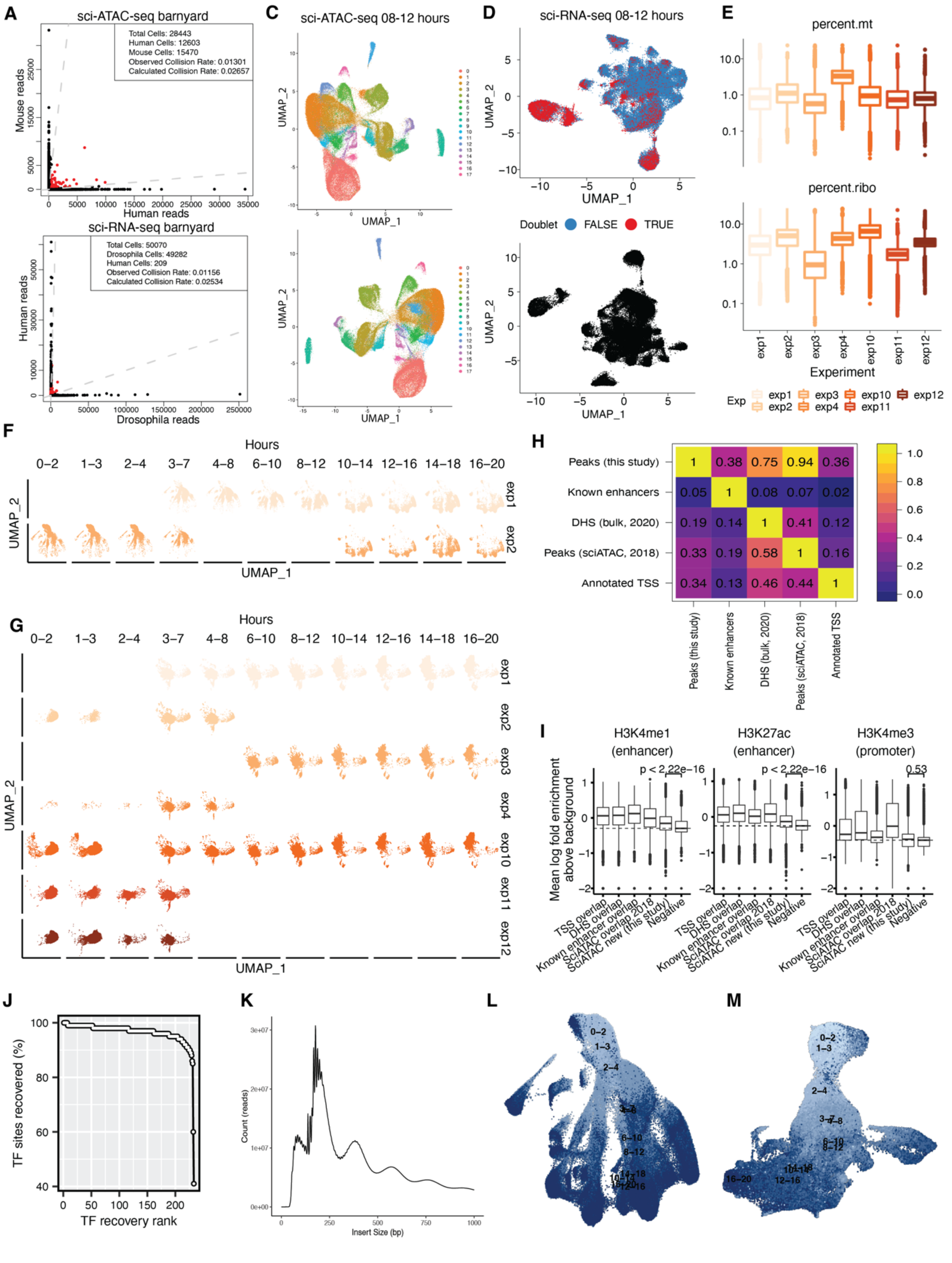

Fig. S2.

Data filtering, evaluating batch and technical effects, and assessing overlap with known enhancers and enhancer-associated biochemical marks. (**A**) Scatterplot showing estimated

doublet rates from a human-mouse barnyard experiment for the scATAC data. (**B**) Scatterplot showing estimated doublet rates from a human-drosophila barnyard experiment for the scRNA data. (**C**) UMAPs for the scATAC data from the 8-12 hr time window before (top) and after (bottom) doublet removal. The clusters are visibly more separated after doublet removal. (**D**) UMAPs for the scRNA cells from the 8-12 hr time window before (top) and after (bottom) doublet removal. (**E**) Barplots of percentage of scRNA reads from ribosomal genes or mitochondria per experiment. (**F**) Global scATAC UMAP faceted by time window and experiment. (**G**) Global scRNA UMAPs faceted by time window and experiment. The lack of UMAP clusters that are specifically deriving from one experiment suggests a lack of strong batch effects. (**H**) Same as **Fig. 1D** but displaying percent overlap of bases between two sets of peaks. (**I**) Log fold enrichment of corresponding ChIP-seq histone modification signal in different peak classes. Newly identified peaks exhibit histone modifications similar to enhancer elements. (**J**) Proportion of TF ChIP-seq binding sites that overlaps with peaks from our data for the full set of 233 TFs included in modERN. (**K**) Insert size distribution across all experiments. (**L**) Same as **Fig. 1B** but including labels of the time window placed at the median of all nuclei collected from this time window. (**M**) Same as **L** but for RNA-seq data.

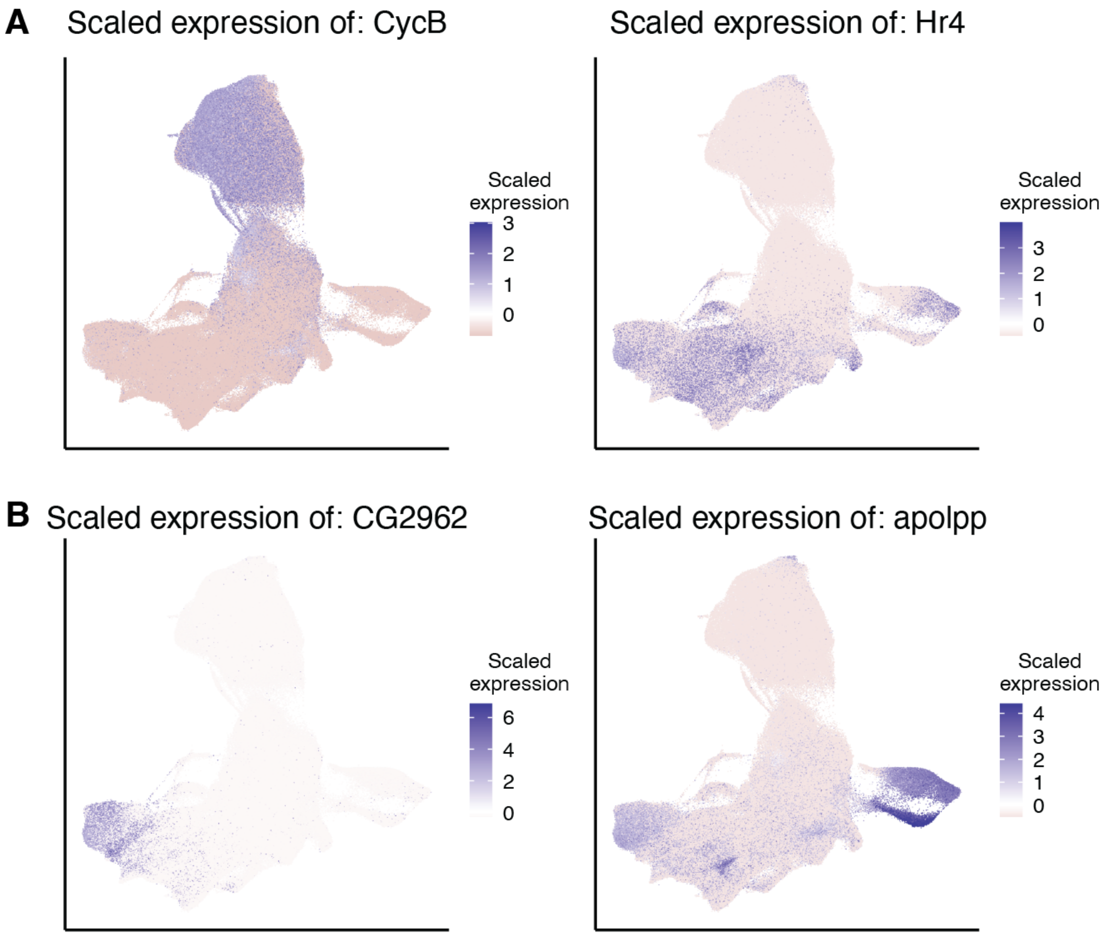

Fig. S3.

Examples of time-dependent genes, some of which appear universal, others of which are specific to certain clusters. (**A**) Two examples of genes that are associated with general expression in cells from early (left) or late (right) time windows. Scaled expression values overlaid on a global UMAP of scRNA data. This is similar to the UMAP shown in **Fig. 1C**, but here with all cells included instead of an evenly time subsampled dataset. (**B**) Similar to panel **A**, except that these time-dependent genes tend to be expressed in later time windows in restricted subsets of UMAP clusters.

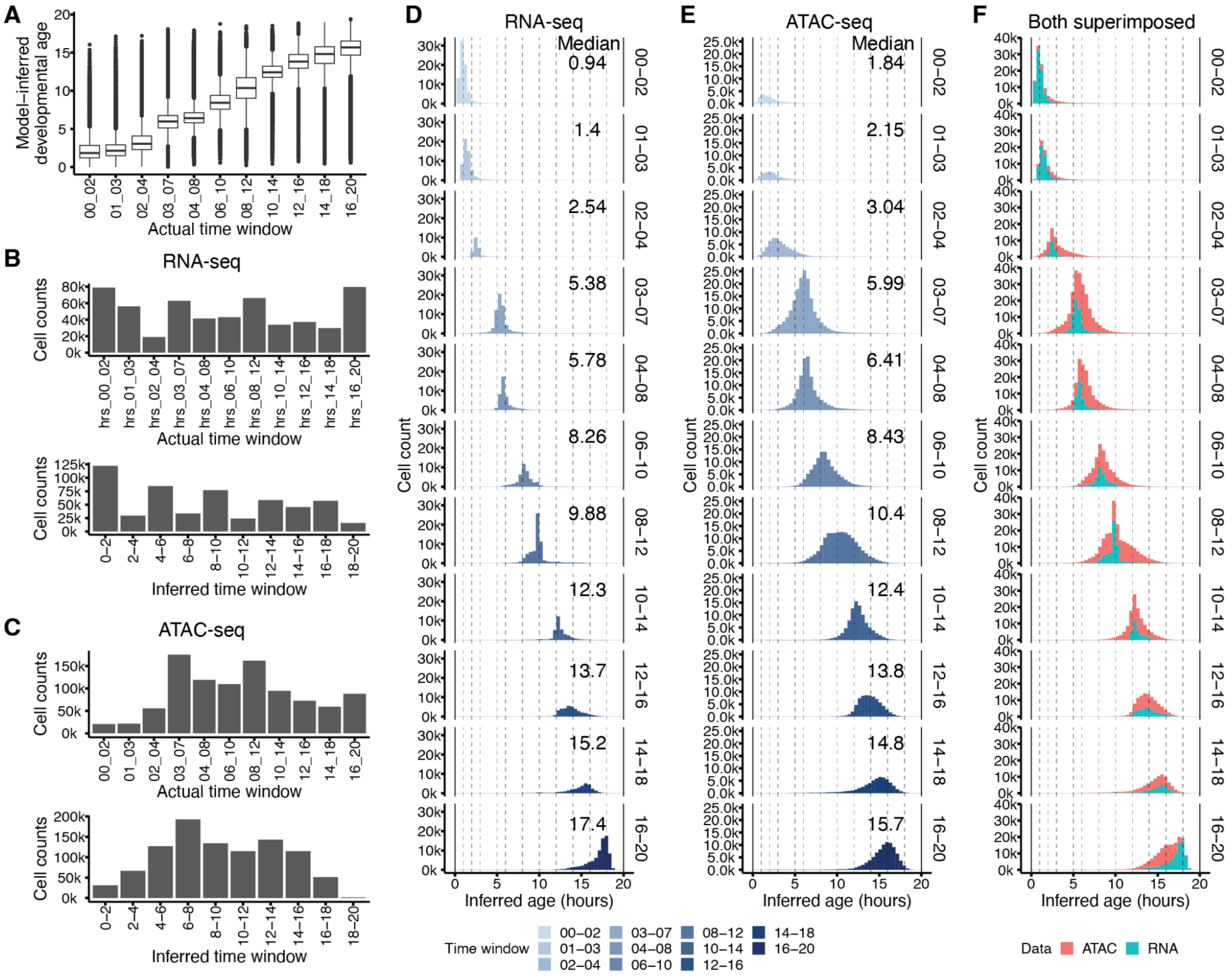

Fig. S4.

A neural network-based model for inferring cellular developmental ages. (**A**) The distribution of model-inferred developmental age for nuclei from the scATAC model across actual time windows (x-axis). (**B**) Counts of cells in scRNA data per actual 2-hr time window (top) and equivalent set of inferred 2-hr time windows (bottom). (**C**) Same as panel **B** except for counts of scATAC nuclei from actual vs. inferred time windows. (**D**) Distribution of inferred ages for cells stratified by the actual time windows from which the cells were collected. Dotted lines correspond to centers of each time window. The median age of cells per actual time window are listed to the right. (**E**) Same as panel **D** but for scATAC data. (**F**) Similar as **E** and **D** but superimposing model-inferred ages from both data types.

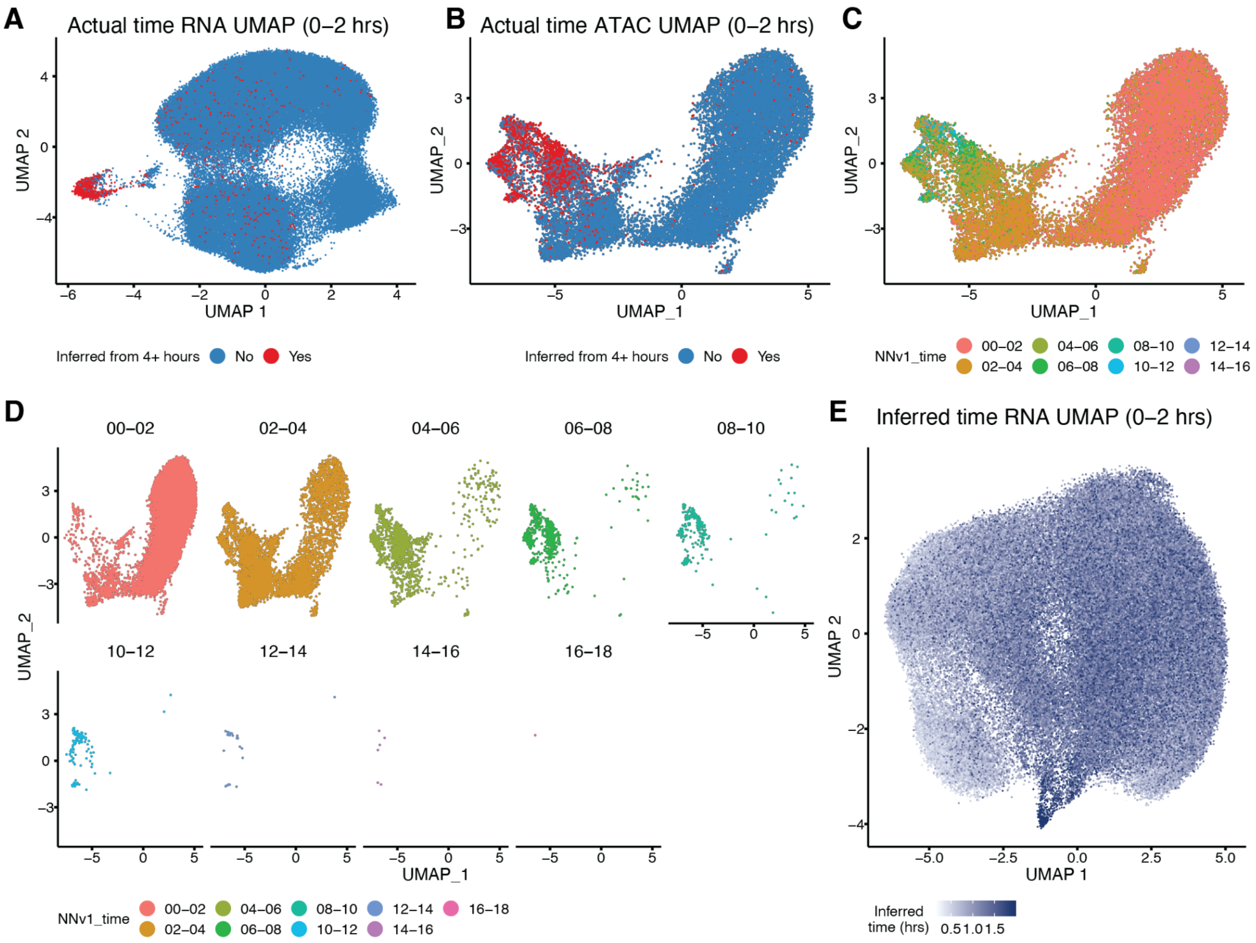

Fig. S5.

Model of developmental ages identifies "contaminating" older nuclei in the earliest collection window. (**A**) UMAP of scRNA data of cells from the 0-2 hr actual time window. Cells inferred to be over 4+ hours in developmental age by the model are highlighted in red. (**B**) Same as panel **A** but visualizing the scATAC data. (**C**) Same as panel **B** but cells are colored by model-inferred time. (**D**) Same as panel **D** but cells are split by model-inferred time. (**E**) UMAP of scRNA data of cells inferred to be up to 2 hrs in developmental age.

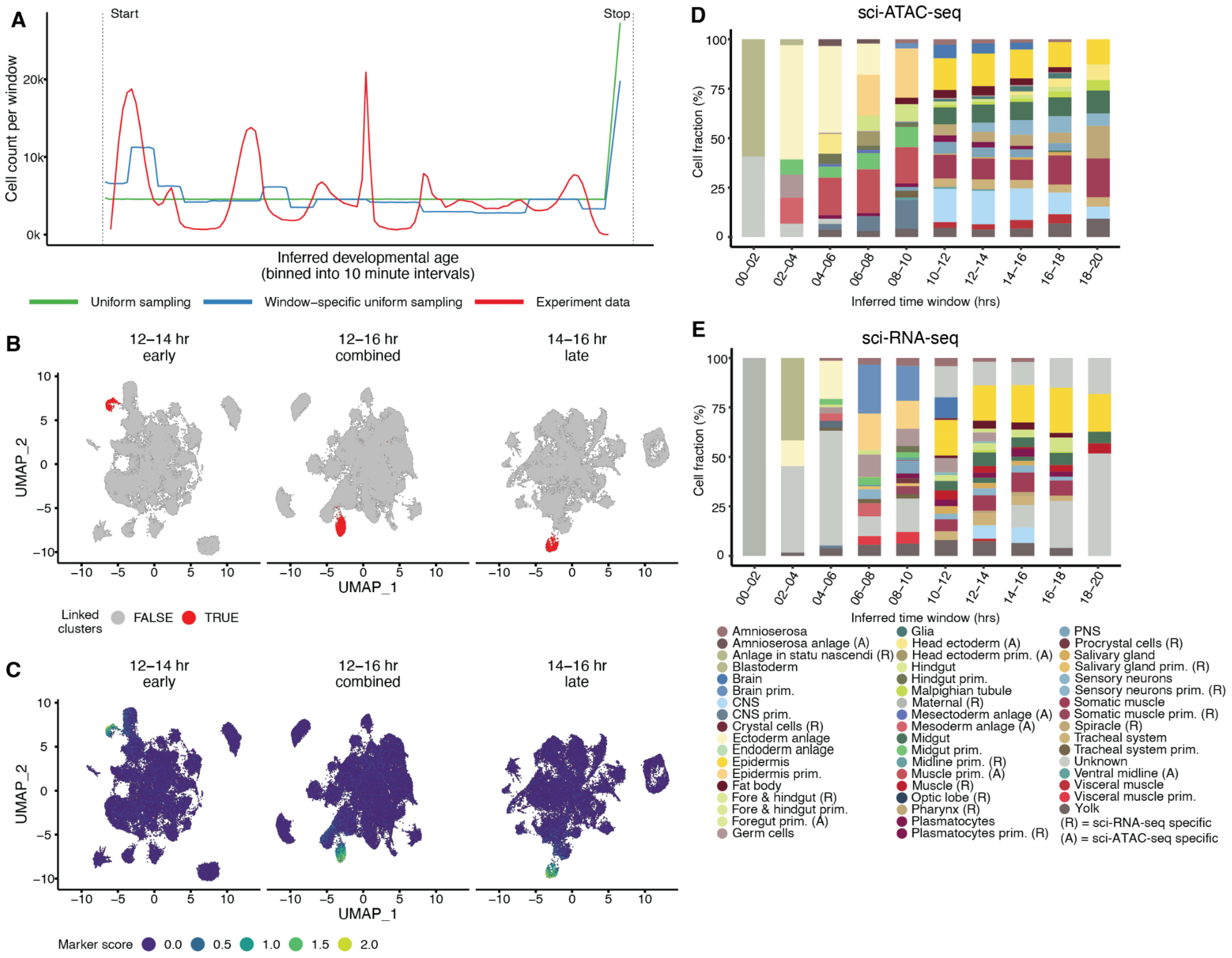

Fig. S6.

Distribution of cells and cell states through inferred time. (**A**) Cells were binned into 10 minute intervals based on their inferred developmental ages. The counts of cells per window are displayed on the y-axis. Counts of cells from the actual distribution of inferred ages for all nuclei are highlighted in red. We wanted to compare the distribution of inferred ages to what might be expected under different potential cell sampling strategies. A uniform sampling of all 547,805 cells across 10 min bins is visualized in green. Alternatively, uniformly sampling cells from actual time windows into 10 min bins is visualized in green. This strategy accounts for different numbers of cells that were collected from different actual time windows. (**B**) Illustration of the process of linking clusters from adjacent time windows. Clusters eventually found to be linked are highlighted in red in adjacent windows (left, right panels), in the co-embedded UMAP space that includes nuclei from both time windows the linked cells are mapped to the same cluster. (**C**) Same UMAP embedding as panel **B,** however cells are colored by their marker gene score defined by marker genes identified from just the 12-14 hr time window linked clustered. (**D**) Cell type proportions in each 2 hour inferred time window of the scATAC data. Cell type colors match those in **Fig. 3A-B**. Cell types annotated as unknown are excluded from the bar graph. (**E**) Same as panel **D** but for the scRNA data.

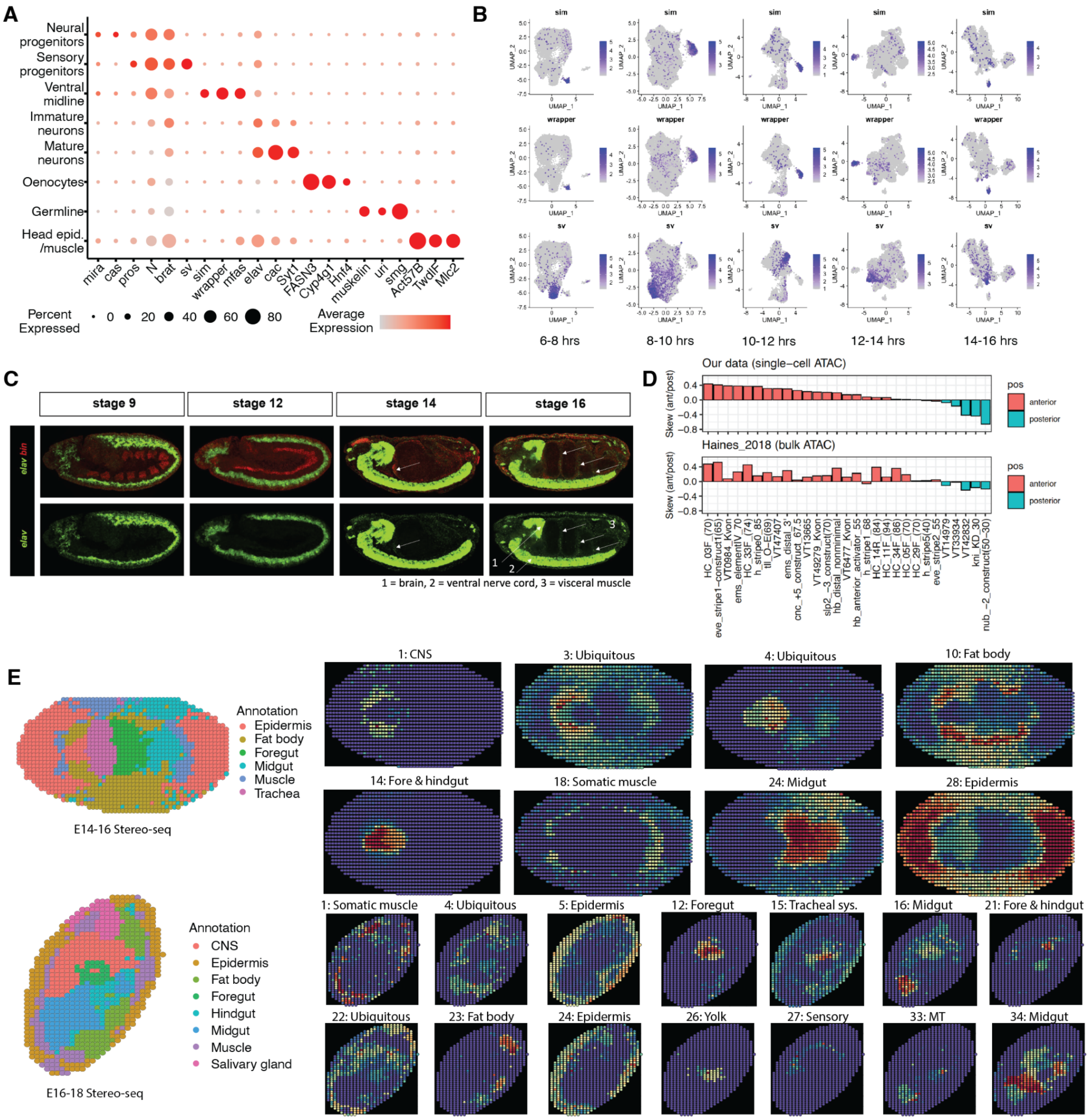

Fig. S7.

Visualizing the expression of neuronal marker genes, unexpected *elav* expression, and spatial analysis. (**A**) Marker gene expression in the neuroectodermal tissues. (**B**) scRNA UMAPs of marker gene expression in the neuroectodermal tissues. (**C**) *In situ* hybridization showing the expression of elav (green) in the nervous system (expected) and in the visceral muscle (unexpected), together with the visceral muscle marker biniou (red). (**D**) Positional accessibility skew ([(anterior - posterior) / total]) at enhancers with validated A-P activity, for aggregated anterior or posterior cells (our data, top panel) and anterior or posterior embryo halves (bulk ATAC-seq from (37), bottom panel). Accessibility is anteriorly skewed for enhancers active in the anterior half of the embryo (light red bars) and posteriorly skewed for

posterior enhancers (light blue bars). (**E**) Reference annotations of *Drosophila* embryos at two time windows (left), and visualization of label transfer probabilities to spatially-located DNA nanoballs (right). The label transfer probabilities are plotted on a blue to red color scale, where large label transfer probabilities are visualized as increasingly red.

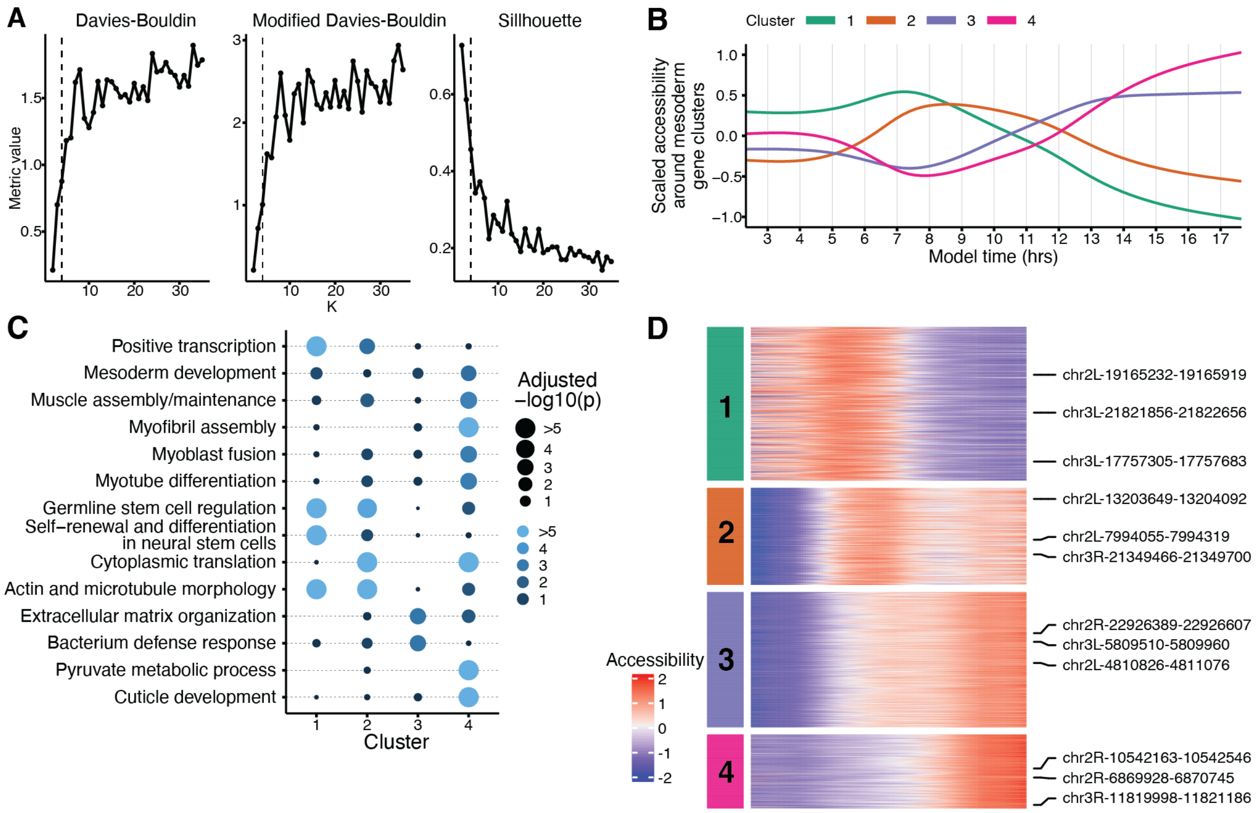

Fig. S8.

Figure S8: Choosing the number of clusters for clustering of variable mesoderm genes, and additional ATAC mesoderm clustering. (**A**) Visualization of several metrics for evaluating different choices of k for clustering. The dotted line indicates our choice of k=4 for clustering mesoderm genes. (**B**) Scaled accessibility around genes that were included in the four mesoderm clusters. (**C**) Results from gene pathway enrichment of genes in the mesoderm-upregulated gene cluster. (**D**) Heatmap of clustered mesoderm accessibility regions using a similar approach as in **Fig. 4C**.

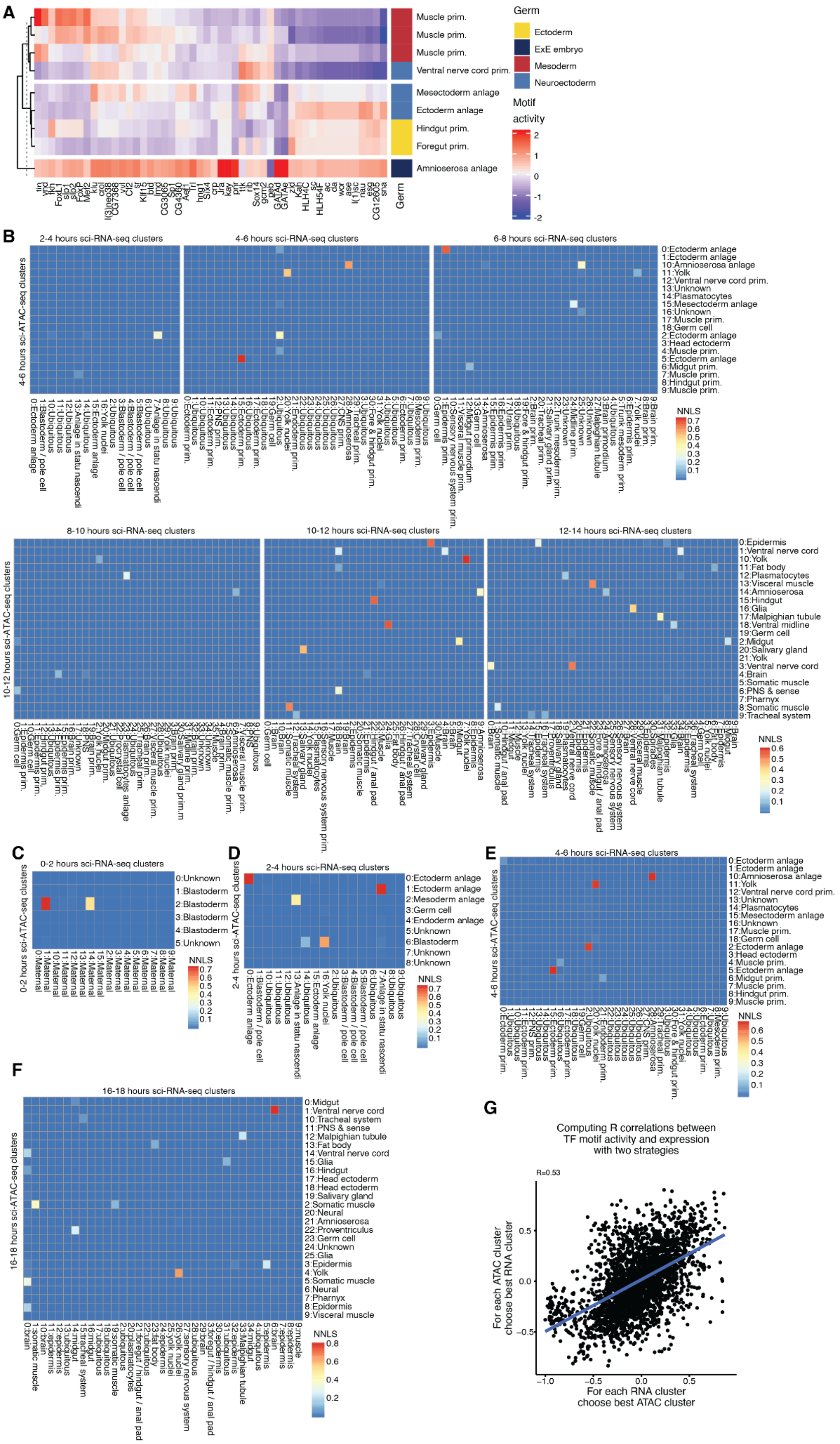
A
Muscle prim.
Muscle prim.
Muscle prim.
Ventral nerve cord prim.
Mesectoderm anlage
Ectoderm anlage
Hindgut prim.
Foregut prim.
Amnioserosa anlage
Germ
Ectoderm
ExE embryo
Mesoderm
Neuroectoderm
Motif activity
2
1
0
-1
-2
B
2-4 hours sci-RNA-seq clusters
4-6 hours sci-RNA-seq clusters
6-8 hours sci-RNA-seq clusters
4-6 hours sci-ATAC-seq clusters
0:Ectoderm anlage
1:Ectoderm anlage
10:Amnioserosa anlage
11:Yolk
12:Ventral nerve cord prim.
13:Unknown
14:Plasmatocytes
15:Mesectoderm anlage
16:Unknown
17:Muscle prim.
18:Germ cell
2:Ectoderm anlage
3:Head ectoderm
4:Muscle prim.
5:Ectoderm anlage
6:Midgut prim.
7:Muscle prim.
8:Hindgut prim.
9:Muscle prim.
NNLS
8-10 hours sci-RNA-seq clusters
10-12 hours sci-RNA-seq clusters
12-14 hours sci-RNA-seq clusters
10-12 hours sci-ATAC-seq clusters
0:Epidermis
1:Ventral nerve cord
10:Yolk
11:Fat body
12:Plasmatocytes
13:Visceral muscle
14:Amnioserosa
15:Hindgut
16:Glia
17:Malpighian tubule
18:Ventral midline
19:Germ cell
2:Midgut
20:Salivary gland
21:Yolk
3:Ventral nerve cord
4:Brain
5:Somatic muscle
6:PNS & sense
7:Pharynx
8:Somatic muscle
9:Tracheal system
C
0-2 hours sci-RNA-seq clusters
0-2 hours sci-ATAC-seq clusters
0:Unknown
1:Blastoderm
2:Blastoderm
3:Blastoderm
4:Blastoderm
5:Unknown
D
2-4 hours sci-RNA-seq clusters
2-4 hours sci-ATAC-seq clusters
0:Ectoderm anlage
1:Ectoderm anlage
2:Mesoderm anlage
3:Germ cell
4:Endoderm anlage
5:Unknown
6:Blastoderm
7:Unknown
8:Unknown
E
4-6 hours sci-RNA-seq clusters
4-6 hours sci-ATAC-seq clusters
F
16-18 hours sci-RNA-seq clusters
16-18 hours sci-ATAC-seq clusters
0:Midgut
1:Ventral nerve cord
10:Tracheal system
11:PNS & sense
12:Malpighian tubule
13:Fat body
14:Ventral nerve cord
15:Glia
16:Hindgut
17:Head ectoderm
18:Head ectoderm
19:Salivary gland
2:Somatic muscle
20:Neural
21:Amnioserosa
22:Proventriculus
23:Germ cell
24:Unknown
25:Glia
3:Epidermis
4:Yolk
5:Somatic muscle
6:Neural
7:Pharynx
8:Epidermis
9:Visceral muscle
G
Computing R correlations between TF motif activity and expression with two strategies
R=0.53
For each ATAC cluster choose best RNA cluster
For each RNA cluster choose best ATAC cluster

Fig. S9.

Motif activity and linking of scATAC and scRNA-derived clusters with non-negative least squares (NNLS). (**A**) Same as **Fig. 5A**, except for the 6-8 hr time window. (**B**) NNLS results for between scATAC annotations and connections to an earlier 2 hr window (left), the matching hour window (middle) and a later 2 hr window of scRNA annotations. (**C-F**) Heatmaps visualizing the NNLS analyses derived coefficients linking scATAC (rows) and scRNA (columns) clusters in several 2 hour inferred time windows. A larger NNLS coefficient indicates higher similarity between the clusters. (**G**) Comparing the Pearson's R correlation values computed between motif-associated chromatin accessibility and TF expression using two distinct strategies of linking ATAC and RNA clusters. On the x-axis, for each RNA cluster we choose the ATAC cluster with the highest NNLS correlation then computed Pearson's R. On the y-axis, for each ATAC cluster we choose the RNA cluster with the highest NNLS correlation. Both strategies resulted in similar correlation values. Blue line indicates a linear regression fit.

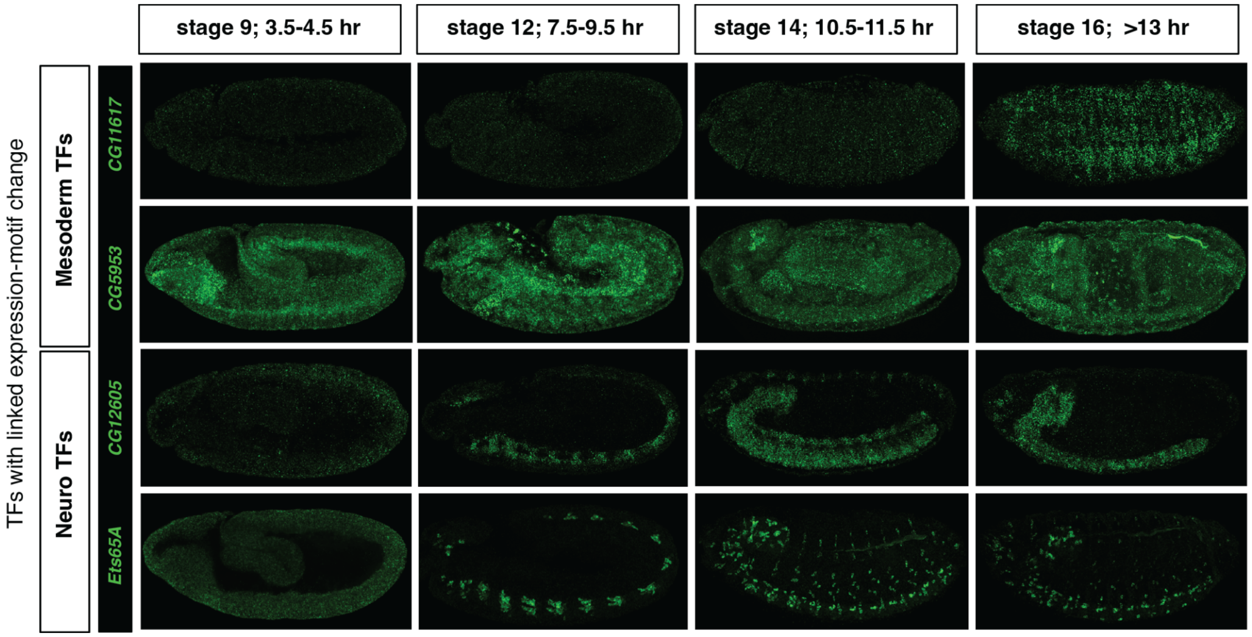

Fig. S10.

Expression validation of putative mesodermal and neuroectodermal TFs by in situ hybridization. *In-situ* hybridization demonstrates the expected temporal and tissue expression of four TFs in the mesoderm / muscle (CG11617, CG5953) or neuroectoderm (CG12605, Ets65A).

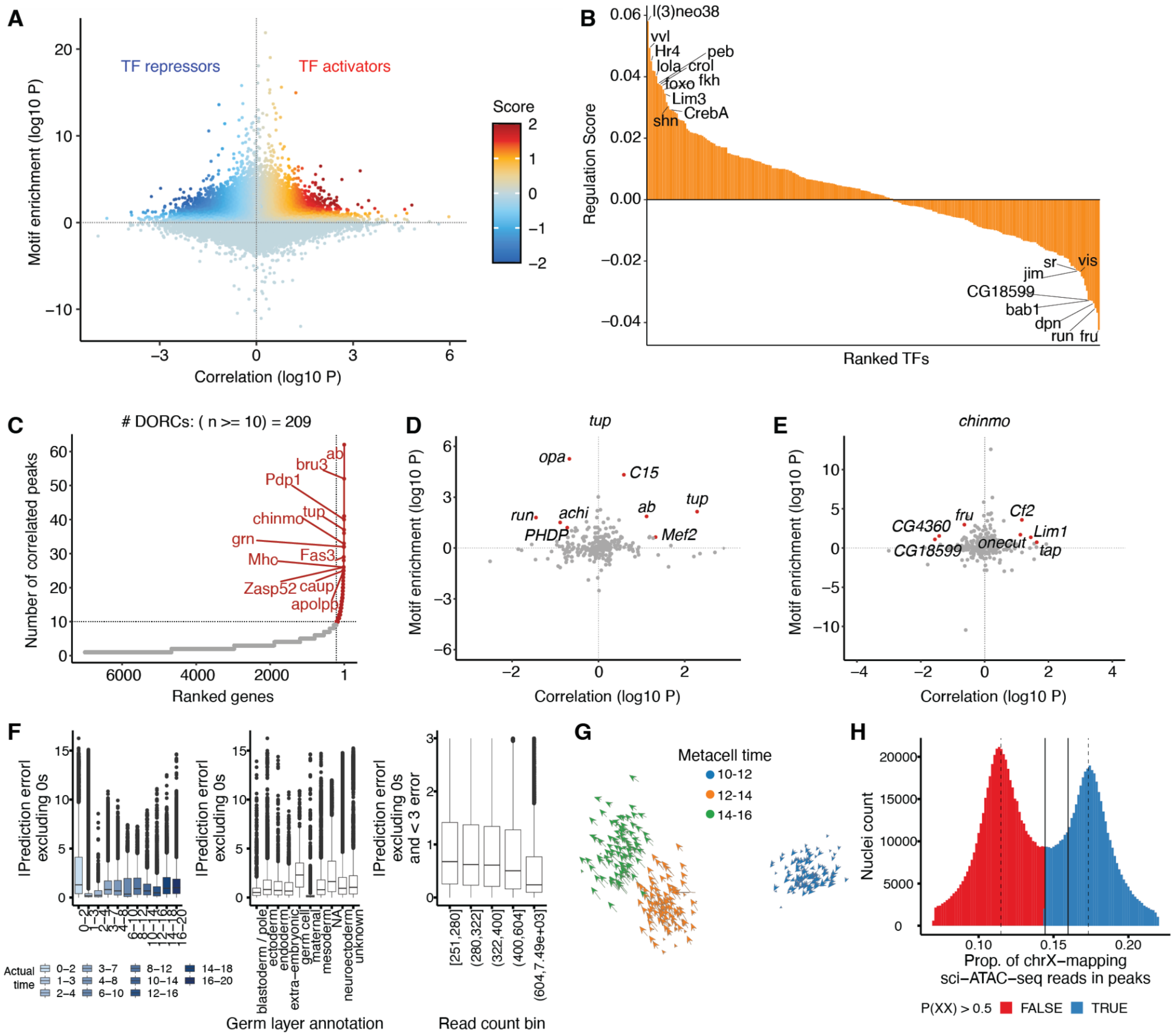

Fig. S11.

Additional analyses of interest. (**A**) Scatterplot of all TF-regulatory domains associations identified by FigR, colored by the regulation score. A positive score indicates putative TF activators, a negative score indicates repressors. (**B**) TFs ranked by their average regulation score (across all associated regulatory regions), putative TF activators (left-skewed) and repressors (right-skewed) are highlighted. (**C**) Genes ranked by the number of significant peak-gene links identified by FigR. Highlighted in red are genes with a high number of peak-gene links (n >= 10), which are referred to as domains of regulatory chromatin (DORCs). (**D**) Candidate TF regulators of *tup*. Highlighted are TFs with an absolute regulation score > 0.5, all other TFs are colored in gray. (**E**) Same as panel **D**, but showing the candidate TF regulators of *chinmo*. (**F**) The error of the model-inferred age prediction is visualized as a boxplot stratified by experimental time window (left), germ layer (middle), or read depth (right). (**G**) The velocity vector estimated on 'metacells', which consisted of a random subsample of cells per window that were pseudobulked prior to velocity analysis. (**H**) Distribution of the proportion of chrX-mapped reads from scATAC data. Cells are classified as XX or XY with a gaussian mixture model.

References and Notes

1. J. Cao, M. Spielmann, X. Qiu, X. Huang, D. M. Ibrahim, A. J. Hill, F. Zhang, S. Mundlos, L. Christiansen, F. J. Steemers, C. Trapnell, J. Shendure, The single-cell transcriptional landscape of mammalian organogenesis. *Nature* **566**, 496–502 (2019). doi:10.1038/s41586-019-0969-x Medline

2. B. Pijuan-Sala, J. A. Griffiths, C. Guibentif, T. W. Hiscock, W. Jawaid, F. J. Calero-Nieto, C. Mulas, X. Ibarra-Soria, R. C. V. Tyser, D. L. L. Ho, W. Reik, S. Srinivas, B. D. Simons, J. Nichols, J. C. Marioni, B. Göttgens, A single-cell molecular map of mouse gastrulation and early organogenesis. *Nature* **566**, 490–495 (2019). doi:10.1038/s41586-019-0933-9 Medline

3. C. Qiu, J. Cao, B. K. Martin, T. Li, I. C. Welsh, S. Srivatsan, X. Huang, D. Calderon, W. S. Noble, C. M. Disteche, S. A. Murray, M. Spielmann, C. B. Moens, C. Trapnell, J. Shendure, Systematic reconstruction of cellular trajectories across mouse embryogenesis. *Nat. Genet.* **54**, 328–341 (2022). doi:10.1038/s41588-022-01018-x Medline

4. M. Mittnenzweig, Y. Mayshar, S. Cheng, R. Ben-Yair, R. Hadas, Y. Rais, E. Chomsky, N. Reines, A. Uzonyi, L. Lumerman, A. Lifshitz, Z. Mukamel, A.-H. Orenbuch, A. Tanay, Y. Stelzer, A single-embryo, single-cell time-resolved model for mouse gastrulation. *Cell* **184**, 2825–2842.e22 (2021). doi:10.1016/j.cell.2021.04.004 Medline

5. G. La Manno, K. Siletti, A. Furlan, D. Gyllborg, E. Vinsland, A. Mossi Albiach, C. Mattsson Langseth, I. Khven, A. R. Lederer, L. M. Dratva, A. Johnsson, M. Nilsson, P. Lönnerberg, S. Linnarsson, Molecular architecture of the developing mouse brain. *Nature* **596**, 92–96 (2021). doi:10.1038/s41586-021-03775-x Medline

6. S. Domcke, A. J. Hill, R. M. Daza, J. Cao, D. R. O'Day, H. A. Pliner, K. A. Aldinger, D. Pokholok, F. Zhang, J. H. Milbank, M. A. Zager, I. A. Glass, F. J. Steemers, D. Doherty, C. Trapnell, D. A. Cusanovich, J. Shendure, A human cell atlas of fetal chromatin accessibility. *Science* **370**, eaba7612 (2020). doi:10.1126/science.aba7612 Medline

7. N. Karaiskos, P. Wahle, J. Alles, A. Boltengagen, S. Ayoub, C. Kipar, C. Kocks, N. Rajewsky, R. P. Zinzen, The *Drosophila* embryo at single-cell transcriptome resolution. *Science* **358**, 194–199 (2017). doi:10.1126/science.aan3235 Medline

8. J. Rivera, S. V. E. Keränen, S. M. Gallo, M. S. Halfon, REDfly: The transcriptional regulatory element database for *Drosophila*. *Nucleic Acids Res.* **47**, D828–D834 (2019). doi:10.1093/nar/gky957 Medline

9. E. Z. Kvon, T. Kazmar, G. Stampfel, J. O. Yáñez-Cuna, M. Pagani, K. Schernhuber, B. J. Dickson, A. Stark, Genome-scale functional characterization of *Drosophila* developmental enhancers *in vivo*. *Nature* **512**, 91–95 (2014). doi:10.1038/nature13395 Medline

10. S. Bonn, R. P. Zinzen, C. Girardot, E. H. Gustafson, A. Perez-Gonzalez, N. Delhomme, Y. Ghavi-Helm, B. Wilczyński, A. Riddell, E. E. M. Furlong, Tissue-specific analysis of chromatin state identifies temporal signatures of enhancer activity during embryonic development. *Nat. Genet.* **44**, 148–156 (2012). doi:10.1038/ng.1064 Medline

11. J. P. Reddington, D. A. Garfield, O. M. Sigalova, A. Karabacak Calviello, R. Marco-Ferreres, C. Girardot, R. R. Viales, J. F. Degner, U. Ohler, E. E. M. Furlong, Lineage-Resolved

Enhancer and Promoter Usage during a Time Course of Embryogenesis. *Dev. Cell* **55**, 648–664.e9 (2020). doi:10.1016/j.devcel.2020.10.009 Medline

12. D. A. Cusanovich, J. P. Reddington, D. A. Garfield, R. M. Daza, D. Aghamirzaie, R. Marco-Ferreres, H. A. Pliner, L. Christiansen, X. Qiu, F. J. Steemers, C. Trapnell, J. Shendure, E. E. M. Furlong, The *cis*-regulatory dynamics of embryonic development at single-cell resolution. *Nature* **555**, 538–542 (2018). doi:10.1038/nature25981 Medline
13. R. P. Zinzen, C. Girardot, J. Gagneur, M. Braun, E. E. M. Furlong, Combinatorial binding predicts spatio-temporal *cis*-regulatory activity. *Nature* **462**, 65–70 (2009). doi:10.1038/nature08531 Medline
14. M. M. Kudron, A. Victorsen, L. Gevirtzman, L. W. Hillier, W. W. Fisher, D. Vafeados, M. Kirkey, A. S. Hammonds, J. Gersch, H. Ammouri, M. L. Wall, J. Moran, D. Steffen, M. Szynkarek, S. Seabrook-Sturgis, N. Jameel, M. Kadaba, J. Patton, R. Terrell, M. Corson, T. J. Durham, S. Park, S. Samanta, M. Han, J. Xu, K.-K. Yan, S. E. Celniker, K. P. White, L. Ma, M. Gerstein, V. Reinke, R. H. Waterston, The ModERN Resource: Genome-Wide Binding Profiles for Hundreds of *Drosophila* and *Caenorhabditis elegans* Transcription Factors. *Genetics* **208**, 937–949 (2018). doi:10.1534/genetics.117.300657 Medline
15. B. R. Graveley, A. N. Brooks, J. W. Carlson, M. O. Duff, J. M. Landolin, L. Yang, C. G. Artieri, M. J. van Baren, N. Boley, B. W. Booth, J. B. Brown, L. Cherbas, C. A. Davis, A. Dobin, R. Li, W. Lin, J. H. Malone, N. R. Mattiuzzo, D. Miller, D. Sturgill, B. B. Tuch, C. Zaleski, D. Zhang, M. Blanchette, S. Dudoit, B. Eads, R. E. Green, A. Hammonds, L. Jiang, P. Kapranov, L. Langton, N. Perrimon, J. E. Sandler, K. H. Wan, A. Willingham, Y. Zhang, Y. Zou, J. Andrews, P. J. Bickel, S. E. Brenner, M. R. Brent, P. Cherbas, T. R. Gingeras, R. A. Hoskins, T. C. Kaufman, B. Oliver, S. E. Celniker, The developmental transcriptome of *Drosophila melanogaster*. *Nature* **471**, 473–479 (2011). doi:10.1038/nature09715 Medline
16. J. C. Kwasnieski, T. L. Orr-Weaver, D. P. Bartel, Early genome activation in *Drosophila* is extensive with an initial tendency for aborted transcripts and retained introns. *Genome Res.* **29**, 1188–1197 (2019). doi:10.1101/gr.242164.118 Medline
17. S. A. Blythe, E. F. Wieschaus, Establishment and maintenance of heritable chromatin structure during early *Drosophila* embryogenesis. *eLife* **5**, e20148 (2016). doi:10.7554/eLife.20148 Medline
18. C. Trapnell, D. Cacchiarelli, J. Grimsby, P. Pokharel, S. Li, M. Morse, N. J. Lennon, K. J. Livak, T. S. Mikkelsen, J. L. Rinn, The dynamics and regulators of cell fate decisions are revealed by pseudotemporal ordering of single cells. *Nat. Biotechnol.* **32**, 381–386 (2014). doi:10.1038/nbt.2859 Medline
19. A. S. Hammonds, C. A. Bristow, W. W. Fisher, R. Weiszmann, S. Wu, V. Hartenstein, M. Kellis, B. Yu, E. Frise, S. E. Celniker, Spatial expression of transcription factors in *Drosophila* embryonic organ development. *Genome Biol.* **14**, R140 (2013). doi:10.1186/gb-2013-14-12-r140 Medline
20. P. Tomancak, B. P. Berman, A. Beaton, R. Weiszmann, E. Kwan, V. Hartenstein, S. E. Celniker, G. M. Rubin, Global analysis of patterns of gene expression during *Drosophila* embryogenesis. *Genome Biol.* **8**, R145 (2007). doi:10.1186/gb-2007-8-7-r145 Medline

21. J. D. Laver, A. J. Marsolais, C. A. Smibert, H. D. Lipshitz, in *Current Topics in Developmental Biology*, vol. 113, H. D. Lipshitz, Ed. (Academic Press, 2015), pp. 43–84.

22. J. A. Briggs, C. Weinreb, D. E. Wagner, S. Megason, L. Peshkin, M. W. Kirschner, A. M. Klein, The dynamics of gene expression in vertebrate embryogenesis at single-cell resolution. *Science* **360**, eaar5780 (2018). doi:10.1126/science.aar5780 Medline

23. W. Song, S. Ressl, W. D. Tracey, Loss of Pseudouridine Synthases in the RluA Family Causes Hypersensitive Nociception in *Drosophila*. *G3* **10**, 4425–4438 (2020). doi:10.1534/g3.120.401767 Medline

24. A. W. Moore, L. Y. Jan, Y. N. Jan, *hamlet*, a binary genetic switch between single- and multiple- dendrite neuron morphology. *Science* **297**, 1355–1358 (2002). doi:10.1126/science.1072387 Medline

25. R. Dubruille, A. Laurençon, C. Vandaele, E. Shishido, M. Coulon-Bublex, P. Swoboda, P. Couble, M. Kernan, B. Durand, *Drosophila* regulatory factor X is necessary for ciliated sensory neuron differentiation. *Development* **129**, 5487–5498 (2002). doi:10.1242/dev.00148 Medline

26. R. G. Walker, A. T. Willingham, C. S. Zuker, A *Drosophila* mechanosensory transduction channel. *Science* **287**, 2229–2234 (2000). doi:10.1126/science.287.5461.2229 Medline

27. J. E. Haines, M. B. Eisen, Patterns of chromatin accessibility along the anterior-posterior axis in the early *Drosophila* embryo. *PLOS Genet.* **14**, e1007367 (2018). doi:10.1371/journal.pgen.1007367 Medline

28. M. Wang, Q. Hu, T. Lv, Y. Wang, Q. Lan, R. Xiang, Z. Tu, Y. Wei, K. Han, C. Shi, J. Guo, C. Liu, T. Yang, W. Du, Y. An, M. Cheng, J. Xu, H. Lu, W. Li, S. Zhang, A. Chen, W. Chen, Y. Li, X. Wang, X. Xu, Y. Hu, L. Liu, High-resolution 3D spatiotemporal transcriptomic maps of developing *Drosophila* embryos and larvae. *Dev. Cell* **57**, 1271–1283.e4 (2022). doi:10.1016/j.devcel.2022.04.006 Medline

29. G. Junion, M. Spivakov, C. Girardot, M. Braun, E. H. Gustafson, E. Birney, E. E. M. Furlong, A transcription factor collective defines cardiac cell fate and reflects lineage history. *Cell* **148**, 473–486 (2012). doi:10.1016/j.cell.2012.01.030 Medline

30. S. Secchia, M. Forneris, T. Heinen, O. Stegle, E. E. M. Furlong, Simultaneous cellular and molecular phenotyping of embryonic mutants using single-cell regulatory trajectories. *Dev. Cell* **57**, 496–511.e8 (2022). doi:10.1016/j.devcel.2022.01.016 Medline

31. G. Frommer, G. Vorbrüggen, G. Pasca, H. Jäckle, T. Volk, Epidermal egr-like zinc finger protein of Drosophila participates in myotube guidance. *EMBO J.* **15**, 1642–1649 (1996). doi:10.1002/j.1460-2075.1996.tb00509.x Medline

32. R. M. Fox, A. Vaishnavi, R. Maruyama, D. J. Andrew, Organ-specific gene expression: The bHLH protein Sage provides tissue specificity to *Drosophila* FoxA. *Development* **140**, 2160–2171 (2013). doi:10.1242/dev.092924 Medline

33. R. Reuter, The gene serpent has homeotic properties and specifies endoderm versus ectoderm within the Drosophila gut. *Development* **120**, 1123–1135 (1994). doi:10.1242/dev.120.5.1123 Medline

34. J. Curtiss, J. S. Heilig, *Arrowhead* encodes a LIM homeodomain protein that distinguishes subsets of *Drosophila* imaginal cells. *Dev. Biol.* **190**, 129–141 (1997). doi:10.1006/dbio.1997.8659 Medline

35. E. Preger-Ben Noon, F. P. Davis, D. L. Stern, Evolved Repression Overcomes Enhancer Robustness. *Dev. Cell* **39**, 572–584 (2016). doi:10.1016/j.devcel.2016.10.010 Medline

36. M. M. Myat, D. J. Andrew, Fork head prevents apoptosis and promotes cell shape change during formation of the Drosophila salivary glands. *Development* **127**, 4217–4226 (2000). doi:10.1242/dev.127.19.4217 Medline

37. V. K. Kartha, F. M. Duarte, Y. Hu, S. Ma, J. G. Chew, C. A. Lareau, A. Earl, Z. D. Burkett, A. S. Kohlway, R. Lebofsky, J. D. Buenrostro, Functional Inference of Gene Regulation using Single-Cell Multi-Omics. bioRxiv 2021.07.28.453784 [Preprint] (2021). https://doi.org/10.1101/2021.07.28.453784.

38. S. A. Blythe, E. F. Wieschaus, Zygotic genome activation triggers the DNA replication checkpoint at the midblastula transition. *Cell* **160**, 1169–1181 (2015). doi:10.1016/j.cell.2015.01.050 Medline

39. M. M. Harrison, X.-Y. Li, T. Kaplan, M. R. Botchan, M. B. Eisen, Zelda binding in the early *Drosophila melanogaster* embryo marks regions subsequently activated at the maternal-to-zygotic transition. *PLOS Genet.* **7**, e1002266 (2011). doi:10.1371/journal.pgen.1002266 Medline

40. J. Duan, L. Rieder, M. M. Colonnetta, A. Huang, M. Mckenney, S. Watters, G. Deshpande, W. Jordan, N. Fawzi, E. Larschan, CLAMP and Zelda function together to promote *Drosophila* zygotic genome activation. *eLife* **10**, e69937 (2021). doi:10.7554/eLife.69937 Medline

41. M. N. Özel, F. Simon, S. Jafari, I. Holguera, Y.-C. Chen, N. Benhra, R. N. El-Danaf, K. Kapuralin, J. A. Malin, N. Konstantinides, C. Desplan, Neuronal diversity and convergence in a visual system developmental atlas. *Nature* **589**, 88–95 (2021). doi:10.1038/s41586-020-2879-3 Medline

42. J. Bageritz, P. Willnow, E. Valentini, S. Leible, M. Boutros, A. A. Teleman, Gene expression atlas of a developing tissue by single cell expression correlation analysis. *Nat. Methods* **16**, 750–756 (2019). doi:10.1038/s41592-019-0492-x Medline

43. C. N. McLaughlin, M. Brbić, Q. Xie, T. Li, F. Horns, S. S. Kolluru, J. M. Kebschull, D. Vacek, A. Xie, J. Li, R. C. Jones, J. Leskovec, S. R. Quake, L. Luo, H. Li, Single-cell transcriptomes of developing and adult olfactory receptor neurons in *Drosophila*. *eLife* **10**, e63856 (2021). doi:10.7554/eLife.63856 Medline

44. Z. Shi, C. Lim, V. Tran, K. Cui, K. Zhao, X. Chen, Single-cyst transcriptome analysis of *Drosophila* male germline stem cell lineage. *Development* **147**, dev.184259 (2020). doi:10.1242/dev.184259 Medline

45. N. J. Everetts, M. I. Worley, R. Yasutomi, N. Yosef, I. K. Hariharan, Single-cell transcriptomics of the *Drosophila* wing disc reveals instructive epithelium-to-myoblast interactions. *eLife* **10**, e61276 (2021). doi:10.7554/eLife.61276 Medline

46. R.-J. Hung, Y. Hu, R. Kirchner, Y. Liu, C. Xu, A. Comjean, S. G. Tattikota, F. Li, W. Song, S. Ho Sui, N. Perrimon, A cell atlas of the adult *Drosophila* midgut. *Proc. Natl. Acad. Sci. U.S.A.* **117**, 1514–1523 (2020). doi:10.1073/pnas.1916820117 Medline

47. H. Li, J. Janssens, M. De Waegeneer, S. S. Kolluru, K. Davie, V. Gardeux, W. Saelens, F. P. A. David, M. Brbić, K. Spanier, J. Leskovec, C. N. McLaughlin, Q. Xie, R. C. Jones, K. Brueckner, J. Shim, S. G. Tattikota, F. Schnorrer, K. Rust, T. G. Nystul, Z. Carvalho-Santos, C. Ribeiro, S. Pal, S. Mahadevaraju, T. M. Przytycka, A. M. Allen, S. F. Goodwin, C. W. Berry, M. T. Fuller, H. White-Cooper, E. L. Matunis, S. DiNardo, A. Galenza, L. E. O'Brien, J. A. T. Dow, FCA Consortium, H. Jasper, B. Oliver, N. Perrimon, B. Deplancke, S. R. Quake, L. Luo, S. Aerts, Fly Cell Atlas: A single-nucleus transcriptomic atlas of the adult fruit fly. *Science* **375**, eabk2432 (2022). doi:10.1126/science.abk2432 Medline

48. J. S. Packer, Q. Zhu, C. Huynh, P. Sivaramakrishnan, E. Preston, H. Dueck, D. Stefanik, K. Tan, C. Trapnell, J. Kim, R. H. Waterston, J. I. Murray, A lineage-resolved molecular atlas of *C. elegans* embryogenesis at single-cell resolution. *Science* **365**, eaax1971 (2019). doi:10.1126/science.aax1971 Medline

49. K. L. Howe, P. Achuthan, J. Allen, J. Allen, J. Alvarez-Jarreta, M. R. Amode, I. M. Armean, A. G. Azov, R. Bennett, J. Bhai, K. Billis, S. Boddu, M. Charkhchi, C. Cummins, L. Da Rin Fioretto, C. Davidson, K. Dodiya, B. El Houdaigui, R. Fatima, A. Gall, C. Garcia Giron, T. Grego, C. Guijarro-Clarke, L. Haggerty, A. Hemrom, T. Hourlier, O. G. Izuogu, T. Juettemann, V. Kaikala, M. Kay, I. Lavidas, T. Le, D. Lemos, J. Gonzalez Martinez, J. C. Marugán, T. Maurel, A. C. McMahon, S. Mohanan, B. Moore, M. Muffato, D. N. Oheh, D. Paraschas, A. Parker, A. Parton, I. Prosovetskaia, M. P. Sakthivel, A. I. A. Salam, B. M. Schmitt, H. Schuilenburg, D. Sheppard, E. Steed, M. Szpak, M. Szuba, K. Taylor, A. Thormann, G. Threadgold, B. Walts, A. Winterbottom, M. Chakiachvili, A. Chaubal, N. De Silva, B. Flint, A. Frankish, S. E. Hunt, G. R. IIsley, N. Langridge, J. E. Loveland, F. J. Martin, J. M. Mudge, J. Morales, E. Perry, M. Ruffier, J. Tate, D. Thybert, S. J. Trevanion, F. Cunningham, A. D. Yates, D. R. Zerbino, P. Flicek, Ensembl 2021. *Nucleic Acids Res.* **49**, D884–D891 (2021). doi:10.1093/nar/gkaa942 Medline

50. T. Sandmann, J. S. Jakobsen, E. E. M. Furlong, ChIP-on-chip protocol for genome-wide analysis of transcription factor binding in *Drosophila melanogaster* embryos. *Nat. Protoc.* **1**, 2839–2855 (2006). doi:10.1038/nprot.2006.383 Medline

51. V. Hartenstein, *Atlas of Drosophila Development* (Cold Spring Harbor Laboratory Press, 1993).

52. S. Bonn, R. P. Zinzen, A. Perez-Gonzalez, A. Riddell, A.-C. Gavin, E. E. M. Furlong, Cell type–specific chromatin immunoprecipitation from multicellular complex samples using BiTS-ChIP. *Nat. Protoc.* **7**, 978–994 (2012). doi:10.1038/nprot.2012.049 Medline

53. I. E. Schor, G. Bussotti, M. Maleš, M. Forneris, R. R. Viales, A. J. Enright, E. E. M. Furlong, Non-coding RNA Expression, Function, and Variation during *Drosophila* Embryogenesis. *Curr. Biol.* **28**, 3547–3561.e9 (2018). doi:10.1016/j.cub.2018.09.026 Medline

54. J. Schindelin, I. Arganda-Carreras, E. Frise, V. Kaynig, M. Longair, T. Pietzsch, S. Preibisch, C. Rueden, S. Saalfeld, B. Schmid, J.-Y. Tinevez, D. J. White, V. Hartenstein, K. Eliceiri, P. Tomancak, A. Cardona, Fiji: An open-source platform for biological-image analysis. *Nat. Methods* **9**, 676–682 (2012). doi:10.1038/nmeth.2019 Medline

55. J. Cao, D. R. O'Day, H. A. Pliner, P. D. Kingsley, M. Deng, R. M. Daza, M. A. Zager, K. A. Aldinger, R. Blecher-Gonen, F. Zhang, M. Spielmann, J. Palis, D. Doherty, F. J. Steemers, I. A. Glass, C. Trapnell, J. Shendure, A human cell atlas of fetal gene expression. *Science* **370**, eaba7721 (2020). doi:10.1126/science.aba7721 Medline

56. T. Stuart, A. Butler, P. Hoffman, C. Hafemeister, E. Papalexi, W. M. Mauck 3rd, Y. Hao, M. Stoeckius, P. Smibert, R. Satija, Comprehensive Integration of Single-Cell Data. *Cell* **177**, 1888–1902.e21 (2019). doi:10.1016/j.cell.2019.05.031 Medline

57. C. S. McGinnis, L. M. Murrow, Z. J. Gartner, DoubletFinder: Doublet Detection in Single-Cell RNA Sequencing Data Using Artificial Nearest Neighbors. *Cell Syst.* **8**, 329–337.e4 (2019). doi:10.1016/j.cels.2019.03.003 Medline

58. P. Tomancak, A. Beaton, R. Weiszmann, E. Kwan, S. Shu, S. E. Lewis, S. Richards, M. Ashburner, V. Hartenstein, S. E. Celniker, G. M. Rubin, Systematic determination of patterns of gene expression during *Drosophila* embryogenesis. *Genome Biol.* **3**, research0088.1 (2002). doi:10.1186/gb-2002-3-12-research0088 Medline

59. A. Sardá-Espinosa, Time-Series Clustering in R Using the dtwclust Package. *R J.* **11**, 22–43 (2019). doi:10.32614/RJ-2019-023

60. C. A. Davis, B. C. Hitz, C. A. Sloan, E. T. Chan, J. M. Davidson, I. Gabdank, J. A. Hilton, K. Jain, U. K. Baymuradov, A. K. Narayanan, K. C. Onate, K. Graham, S. R. Miyasato, T. R. Dreszer, J. S. Strattan, O. Jolanki, F. Y. Tanaka, J. M. Cherry, The Encyclopedia of DNA elements (ENCODE): Data portal update. *Nucleic Acids Res.* **46**, D794–D801 (2018). doi:10.1093/nar/gkx1081 Medline

61. E. Y. Chen, C. M. Tan, Y. Kou, Q. Duan, Z. Wang, G. V. Meirelles, N. R. Clark, A. Ma'ayan, Enrichr: Interactive and collaborative HTML5 gene list enrichment analysis tool. *BMC Bioinformatics* **14**, 128 (2013). doi:10.1186/1471-2105-14-128 Medline

62. M. V. Kuleshov, J. E. L. Diaz, Z. N. Flamholz, A. B. Keenan, A. Lachmann, M. L. Wojciechowicz, R. L. Cagan, A. Ma'ayan, modEnrichr: A suite of gene set enrichment analysis tools for model organisms. *Nucleic Acids Res.* **47**, W183–W190 (2019). doi:10.1093/nar/gkz347 Medline

63. V. Bergen, M. Lange, S. Peidli, F. A. Wolf, F. J. Theis, Generalizing RNA velocity to transient cell states through dynamical modeling. *Nat. Biotechnol.* **38**, 1408–1414 (2020). doi:10.1038/s41587-020-0591-3 Medline

64. M. R. Corces, A. E. Trevino, E. G. Hamilton, P. G. Greenside, N. A. Sinnott-Armstrong, S. Vesuna, A. T. Satpathy, A. J. Rubin, K. S. Montine, B. Wu, A. Kathiria, S. W. Cho, M. R. Mumbach, A. C. Carter, M. Kasowski, L. A. Orloff, V. I. Risca, A. Kundaje, P. A. Khavari, T. J. Montine, W. J. Greenleaf, H. Y. Chang, An improved ATAC-seq protocol reduces background and enables interrogation of frozen tissues. *Nat. Methods* **14**, 959–962 (2017). doi:10.1038/nmeth.4396 Medline

65. Y. Zhang, T. Liu, C. A. Meyer, J. Eeckhoute, D. S. Johnson, B. E. Bernstein, C. Nusbaum, R. M. Myers, M. Brown, W. Li, X. S. Liu, Model-based analysis of ChIP-Seq (MACS). *Genome Biol.* **9**, R137 (2008). doi:10.1186/gb-2008-9-9-r137 Medline

66. A. R. Quinlan, I. M. Hall, BEDTools: A flexible suite of utilities for comparing genomic features. *Bioinformatics* **26**, 841–842 (2010). doi:10.1093/bioinformatics/btq033 Medline

67. S. L. Wolock, R. Lopez, A. M. Klein, Scrublet: Computational Identification of Cell Doublets in Single-Cell Transcriptomic Data. *Cell Syst.* **8**, 281–291.e9 (2019). doi:10.1016/j.cels.2018.11.005 Medline

68. T. Stuart, A. Srivastava, C. Lareau, R. Satija, Multimodal single-cell chromatin analysis with Signac. bioRxiv 2020.11.09.373613 [Preprint] (2020). https://doi.org/10.1101/2020.11.09.373613.

69. M. T. Weirauch, A. Yang, M. Albu, A. G. Cote, A. Montenegro-Montero, P. Drewe, H. S. Najafabadi, S. A. Lambert, I. Mann, K. Cook, H. Zheng, A. Goity, H. van Bakel, J.-C. Lozano, M. Galli, M. G. Lewsey, E. Huang, T. Mukherjee, X. Chen, J. S. Reece-Hoyes, S. Govindarajan, G. Shaulsky, A. J. M. Walhout, F.-Y. Bouget, G. Ratsch, L. F. Larrondo, J. R. Ecker, T. R. Hughes, Determination and inference of eukaryotic transcription factor sequence specificity. *Cell* **158**, 1431–1443 (2014). doi:10.1016/j.cell.2014.08.009 Medline

70. A. N. Schep, B. Wu, J. D. Buenrostro, W. J. Greenleaf, chromVAR: Inferring transcription-factor-associated accessibility from single-cell epigenomic data. *Nat. Methods* **14**, 975–978 (2017). doi:10.1038/nmeth.4401 Medline

71. S. Heinz, C. Benner, N. Spann, E. Bertolino, Y. C. Lin, P. Laslo, J. X. Cheng, C. Murre, H. Singh, C. K. Glass, Simple combinations of lineage-determining transcription factors prime *cis*-regulatory elements required for macrophage and B cell identities. *Mol. Cell* **38**, 576–589 (2010). doi:10.1016/j.molcel.2010.05.004 Medline

72. J. Friedman, T. Hastie, R. Tibshirani, Regularization Paths for Generalized Linear Models via Coordinate Descent. *J. Stat. Softw.* **33**, 1–22 (2010). doi:10.18637/jss.v033.i01 Medline

73. M. Abadi, A. Agarwal, P. Barham, E. Brevdo, Z. Chen, C. Citro, G. S. Corrado, A. Davis, J. Dean, M. Devin, S. Ghemawat, I. Goodfellow, A. Harp, G. Irving, M. Isard, Y. Jia, R. Jozefowicz, L. Kaiser, M. Kudlur, J. Levenberg, D. Mane, R. Monga, S. Moore, D. Murray, C. Olah, M. Schuster, J. Shlens, B. Steiner, I. Sutskever, K. Talwar, P. Tucker, V. Vanhoucke, V. Vasudevan, F. Viegas, O. Vinyals, P. Warden, M. Wattenberg, M. Wicke, Y. Yu, X. Zheng, TensorFlow: Large-Scale Machine Learning on Heterogeneous Distributed Systems. arXiv:1603.04467 [cs.DC] (2016).

74. M. Abadi, P. Barham, J. Chen, Z. Chen, A. Davis, J. Dean, M. Devin, S. Ghemawat, G. Irving, M. Isard, M. Kudlur, J. Levenberg, R. Monga, S. Moore, D. G. Murray, B. Steiner, P. Tucker, V. Vasudevan, P. Warden, M. Wicke, Y. Yu, X. Zheng, in *OSDI'16: Proceedings of the 12th USENIX Conference on Operating Systems Design and Implementation* (USENIX Association, 2016), pp. 265–283.

75. T. Benaglia, D. Chauveau, D. R. Hunter, D. S. Young, mixtools: An R Package for Analyzing Mixture Models. *J. Stat. Softw.* **32**, 1–29 (2009). doi:10.18637/jss.v032.i06

76. C. Qiu, J. Cao, T. Li, S. Srivatsan, X. Huang, D. Calderon, W. S. Noble, C. M. Disteche, M. Spielmann, C. B. Moens, C. Trapnell, J. Shendure, Systematic reconstruction of the cellular trajectories of mammalian embryogenesis. bioRxiv 2021.06.08.447626 [Preprint] (2021). https://doi.org/10.1101/2021.06.08.447626.

77. D. K. Hoshizaki, T. Blackburn, C. Price, M. Ghosh, K. Miles, M. Ragucci, R. Sweis, Embryonic fat-cell lineage in Drosophila melanogaster. *Development* **120**, 2489–2499 (1994). doi:10.1242/dev.120.9.2489 Medline

78. B. de Velasco, L. Mandal, M. Mkrtchyan, V. Hartenstein, Subdivision and developmental fate of the head mesoderm in *Drosophila melanogaster*. *Dev. Genes Evol.* **216**, 39–51 (2006). doi:10.1007/s00427-005-0029-4 Medline

79. W. Wood, A. Jacinto, *Drosophila melanogaster* embryonic haemocytes: Masters of multitasking. *Nat. Rev. Mol. Cell Biol.* **8**, 542–551 (2007). doi:10.1038/nrm2202 Medline

80. N. C. Franc, P. Heitzler, R. A. B. Ezekowitz, K. White, Requirement for croquemort in phagocytosis of apoptotic cells in *Drosophila*. *Science* **284**, 1991–1994 (1999). doi:10.1126/science.284.5422.1991 Medline

81. J. Manaka, T. Kuraishi, A. Shiratsuchi, Y. Nakai, H. Higashida, P. Henson, Y. Nakanishi, Draper-mediated and phosphatidylserine-independent phagocytosis of apoptotic cells by *Drosophila* hemocytes/macrophages. *J. Biol. Chem.* **279**, 48466–48476 (2004). doi:10.1074/jbc.M408597200 Medline

82. I. Vlisidou, W. Wood, *Drosophila* blood cells and their role in immune responses. *FEBS J.* **282**, 1368–1382 (2015). doi:10.1111/febs.13235 Medline

83. R. Ugrankar, J. Bowerman, H. Hariri, M. Chandra, K. Chen, M.-F. Bossanyi, S. Datta, S. Rogers, K. M. Eckert, G. Vale, A. Victoria, J. Fresquez, J. G. McDonald, S. Jean, B. M. Collins, W. M. Henne, *Drosophila* Snazarus Regulates a Lipid Droplet Population at Plasma Membrane-Droplet Contacts in Adipocytes. *Dev. Cell* **50**, 557–572.e5 (2019). doi:10.1016/j.devcel.2019.07.021 Medline

84. M. Rodríguez-Vázquez, D. Vaquero, E. Parra-Peralbo, J. E. Mejía-Morales, J. Culi, *Drosophila* Lipophorin Receptors Recruit the Lipoprotein LTP to the Plasma Membrane to Mediate Lipid Uptake. *PLOS Genet.* **11**, e1005356 (2015). doi:10.1371/journal.pgen.1005356 Medline

85. E. Demir, B. J. Dickson, *fruitless* splicing specifies male courtship behavior in *Drosophila*. *Cell* **121**, 785–794 (2005). doi:10.1016/j.cell.2005.04.027 Medline

86. D. S. Manoli, M. Foss, A. Villella, B. J. Taylor, J. C. Hall, B. S. Baker, Male-specific *fruitless* specifies the neural substrates of *Drosophila* courtship behaviour. *Nature* **436**, 395–400 (2005). doi:10.1038/nature03859 Medline

87. K. Kimura, M. Ote, T. Tazawa, D. Yamamoto, Fruitless specifies sexually dimorphic neural circuitry in the *Drosophila* brain. *Nature* **438**, 229–233 (2005). doi:10.1038/nature04229 Medline

88. M. V. Brovkina, R. Duffié, A. E. C. Burtis, E. J. Clowney, Fruitless decommissions regulatory elements to implement cell-type-specific neuronal masculinization. *PLOS Genet.* **17**, e1009338 (2021). doi:10.1371/journal.pgen.1009338 Medline

89. K. Sato, D. Yamamoto, The mode of action of Fruitless: Is it an easy matter to switch the sex? *Genes Brain Behav.* **19**, e12606 (2020). doi:10.1111/gbb.12606 Medline

90. H. Ito, K. Sato, S. Kondo, R. Ueda, D. Yamamoto, Fruitless Represses *robo1* Transcription to Shape Male-Specific Neural Morphology and Behavior in *Drosophila*. *Curr. Biol.* **26**, 1532–1542 (2016). doi:10.1016/j.cub.2016.04.067 Medline
