# Supplementary material for: The continuum of Drosophila embryonic development at single-cell resolution
Source: Science. Author manuscript; Available in PMC 2022 Aug 11. (PMC9371440; doi:10.1126/science.abn5800)
Supplement: Supplementary tables [file NIHMS1828489-supplement-Supplementary_tables.zip › science.abn5800_table_captions.pdf]

Additional Data Table S1 (separate file)
RNA cell type annotations. For each cluster in each time window of the RNA-seq data we provide an automated cell type or tissue annotation, a manual cell type annotation that incorporates information from marker genes and germ layer.

Additional Data Table S2 (separate file)
ATAC cell type annotations. For each cluster in each time window of the ATAC-seq data we provide an automated cell type annotation based on integration with previously annotated datasets, a refined cell type annotation that incorporates information from marker regions, marker genes and germ layer, and lineage information based on connecting the clusters from neighboring time windows.

Additional Data Table S3 (separate file)
RNA marker genes. Selected marker genes for each cluster in each time window of the RNA-seq data identified with a differential accessibility test with high statistical significance

Additional Data Table S4 (separate file)
ATAC marker accessible regions. Selected marker regions for each cluster in each time window of the ATAC-seq data identified with a differential accessibility test with high statistical significance.

Additional Data Table S5 (separate file)
Marker genes that differentiate each identified cluster in the neuronal data subset. Selected marker genes for each sci-RNA-seq cell state from the neuroectodermal vignette in Fig. 3, identified with a differential accessibility test with high statistical significance. Marker genes for the clustering of the full neuroectodermal set of cells are indicated by the 'Clustering' field set to 'Broad', whereas marker genes for the clustering of just the mature neuron annotated set of cells are indicated by the 'Clustering' field set to 'Fine'.

Additional Data Table S6 (separate file)
Variably expressed mesoderm genes and their cluster assignment. The set of variably expressed genes identified in the mesoderm-annotated cell types which were then grouped into clusters by their patterns of temporal co-regulation.

Additional Data Table S7 (separate file)
TF motifs enriched in accessible regions near genes from mesoderm gene clusters. Results from the home-based motif enrichment in accessible regions that were proximal to the variably expressed mesoderm genes. These values are separated into the different mesoderm cluster groups.

Additional Data Table S8 (separate file)
NNLS-based links between RNA and ATAC clusters. NNLS coefficients for each pair of clusters of the ATAC-seq data and the RNA-seq data separated by the different 2 hr time windows.

Additional Data Table S9 (separate file)
Correlations between motif and expression. For each of the TFs with a motif in cisBP we computed the Pearson correlation between the expression of the TF and associated motif

accessibility score. These values were computed at every time window.

Additional Data Table S10 (separate file)
Model effect estimates between motif and expression in germ layers by time. Linear regression effect estimates comparing TF expression with its corresponding motif accessibility score with a model that includes an interaction term that includes germ layer, time window.

Additional Data Table S11 (separate file)
UMAP clustering resolutions per time window for RNA and ATAC. For each time window we performed clustering of the data with multiple resolution parameter values then selected the parameter value at which the variability explained of the resulting clusters plateaued.

Additional Data Table S12 (separate file)
Variably accessible mesoderm peaks and their cluster assignment. The set of variably expressed accessible regions identified in the mesoderm-annotated cell types which were then grouped into clusters by their patterns of temporal co-regulation.

Additional Data Table S13 (separate file)
TF motifs enriched in mesoderm accessible region clusters. Results from the home-based motif enrichment in the accessible regions that were variable in mesoderm-annotated cells. These values are separated into the different mesoderm cluster groups.
